# Preparing tomorrow’s health entrepreneurs: a collaborative multi-stakeholder approach to identifying core competencies and training needs of future professionals

**DOI:** 10.1186/s12913-025-13571-2

**Published:** 2025-10-28

**Authors:** Edgar Mascarenhas, Filomena Carnide, Mónica D. Oliveira

**Affiliations:** 1 CEGIST, Instituto Superior Técnico, Universidade de Lisboa, Av. Rovisco Pais, 1, 1049 - 001 Lisboa, Lisbon, Portugal; 2 LBMF, CIPER, Faculdade de Motricidade Humana, Universidade de Lisboa, Instituto Superior Técnico, Universidade de Lisboa, Estrada da Costa, 1499-002 Cruz Quebrada-Dafundo, Lisbon, Portugal; 3 iBB- Institute for Bioengineering and Biosciences and i4HB- Associate Laboratory Institute for Health and Bioeconomy, Instituto Superior Técnico, Universidade de Lisboa, Av. Rovisco Pais, 1, 1049 - 001 Lisboa, Lisbon, Portugal

**Keywords:** Health entrepreneurship, Innovation, Competencies, Health education, Curriculum development, Multi-stakeholder engagement, Scoping review, Delphi method, Workshop, Value-focused thinking

## Abstract

**Background:**

Health systems face complex and multifaceted challenges that require health professionals (including clinicians, nurses and other health workers) to be equipped with entrepreneurship and innovation (E&I) competencies. However, the integration of E&I training within health university curricula remains largely underexplored across different countries and educational contexts. This study aimed to identify core E&I competencies and aligned training needs for future health professionals through a structured, multi-stakeholder approach.

**Methods:**

Shaped within a value-focused thinking framework (systematically linking competencies as end goals with educational topics as means to achieve competencies), the study comprised three stages: (1) a scoping review (ScR) combined with qualitative content analysis (QCA) to identify and categorise E&I competencies and course topics documented in literature, alongside a value-driven competencies-topics relevance matrix; (2) a 3-round web-Delphi process involving a diverse panel of health stakeholders who validated and refined competencies and course topics lists (presented as pre-built thematic maps within the web-Delphi platform), and assessed course topics’ relevance for developing specific E&I competencies; (3) a facilitated virtual workshop to discuss Delphi results and implications for higher education.

**Results:**

The ScR included twenty-nine studies. QCA of these studies produced initial lists of 28 competencies (grouped into five E&I domains of competency) and 34 course topics, organized into thematic maps. Through the responses of 29 Delphi participants, these lists were expanded to a final set of 51 competencies and 55 competency-aligned course topics. Participants rated 91% of topics as at least strongly relevant for developing the respective E&I domain of competency. The workshop provided insights on implementing formal E&I training within health education curricula, with specific reference to the Portuguese context.

**Conclusions:**

Results provide educational institutions with a practical starting point for reflecting on and designing profession-specific E&I courses and enhancing existing graduate programs through targeted E&I competency integration. Findings have significant implications for health workforce education and planning, particularly in promoting competency-driven E&I curricula to enhance professionals’ readiness for current and future healthcare system challenges. This study presents an innovative multi-stakeholder collaborative approach for mapping E&I competencies and aligned topics for training health professionals.

**Supplementary Information:**

The online version contains supplementary material available at 10.1186/s12913-025-13571-2.

## Introduction

Modern health systems face numerous complex and interrelated challenges. Demographic transitions, ageing populations and the burden of chronic diseases are increasing the demand for high-quality and timely healthcare provision, while constrained budgets struggle to keep up with escalating healthcare expenditures [1]. The rapid advancement of technological innovations, including digital health and artificial intelligence (AI) tools, are transforming the delivery, management, and financing of health services [2–4], whilst simultaneously raising ethical and legal concerns that warrant careful consideration [5]. Furthermore, climate change presents an emerging global health threat [6] that is anticipated to exacerbate health risks and intensify global inequalities [7].

Effectively addressing these and other challenges requires a systemic transformation of health systems worldwide. Health professionals – including physicians, nurses, therapists, health technicians, among others – play a pivotal role in this transformation; however, their capacity to contribute meaningfully is contingent upon their preparedness to understand and effectively navigate the complexities of the healthcare landscape [8, 9].

Previous research has highlighted the urgent need to redesign health education curricula in higher education [10–13], as traditional approaches are typically fragmented and static, with a narrow focus on technical and clinical knowledge and skills [14, 15]. While these competencies remain essential, they may be insufficient to fully prepare health workforce for the modern-day health system challenges [16]. Indeed, it is often posited that there is a mismatch between professional competencies in health care workforce and their training requirements in an increasingly interdependent world [10, 17, 18].

A more comprehensive health education framework is therefore required – one that integrates entrepreneurship and innovation (E&I) competencies into the core of health education [19] – thereby equipping health professionals with the appropriate knowledge, skills, and attitudes, so that they are able to seize opportunities, drive systemic change, and develop innovative and sustainable solutions, ultimately shaping the future of healthcare [20–22].

While the importance of E&I training within health education has been increasingly acknowledged [e.g. 23, 24], the incorporation of E&I-related content into health education curricula remains largely underdeveloped [25]. Where these topics are included, they are frequently treated as ancillary rather than integral to professional development [26]. This gap is problematic, as it leaves health professionals without the competencies needed to act as agents of change in their organizations and beyond [27, 28].

The integration of formal and systematic E&I training in health-related education programmes is of paramount importance. However, effective implementation requires more than the mere introduction of generic E&I concepts into existing curricula. To maximise the impact of E&I training in health education, it is essential to first obtain a comprehensive understanding of the set of entrepreneurial competencies and skills a health professional must possess in today’s complex work environment. To achieve this objective, the active involvement of health professionals and relevant stakeholders in this process is crucial to ensure a more practically relevant and impactful competency-based education [29–31] for informing the development of a curriculum closely aligned with the real-world challenges and evolving demands of the health sector [32].

Furthermore, significant gaps remain in understanding how to achieve constructive alignment [33] in E&I health education curricula, linking E&I core competencies relevant for health professionals with educational training needs [34, 35]. Much of the existing literature focuses on general competency frameworks [e.g. 36, 37] or isolated case studies describing training programs and curricula [e.g. 38, 39], often without explicitly providing a direct correspondence between these two highly interdependent (and thus not mutually exclusive) components: competencies and curricular needs. This lack of alignment between what students should achieve (competencies) and how curricula are designed to help them achieve such competencies (course topics and learning activities) represents a critical gap in E&I health education design [40].

This study seeks to address these gaps by conducting a structured, multi-stakeholder collaborative approach, engaging experts and professionals within the health sector to identify core E&I competencies and training needs (in the form of course topics) deemed relevant to contribute to development of those E&I competencies. Specifically, the primary objective of this study is to contribute to the E&I in health literature by providing a comprehensive, stakeholder-validated framework that bridges competencies and educational content for practical curriculum development, thus proving a starting point for the discussion of how E&I training can be adequately included in higher education health programmes and courses. As a secondary (methodological) contribution, we demonstrate how the use of visual and interactive elements can be useful in facilitating participant understanding of complex relationships between different constructs (competencies, course topics) and promoting engagement within web-based formal collaborative processes.

To accomplish these objectives, this study comprises a scoping review of the literature, a modified three-round web-based Delphi process and a final virtual workshop. By applying a Value-Focused Thinking (VFT) [41] approach throughout all these stages, we attempt to link these two interconnected components, thereby considering competencies as *end goals* (i.e. what is to be achieved) and topics as *means* to achieve those goals (i.e. how one will achieve it). E&I competencies are taken as the *integrated combination of knowledge*,* skills*,* attitudes (KSA) that enable health professionals to identify opportunities*,* develop innovative solutions*,* and drive transformative change within health systems*. This definition is grounded in Bloom’s taxonomy of educational goals [42], which categorizes learning into cognitive (knowledge), psychomotor (skills), and affective (attitudes) domains.

This study was conducted under the scope of the HEI4Future European Union (EU) project [43] (grant agreement number: 23419) – funded by the EU under the scope of the EIT HEI Initiative [44] – and aimed at developing entrepreneurial and innovation skills within higher education institutions across Europe by designing and developing a set of innovative and multi-disciplinary activities to reinforce higher education training on E&I, contributing to bridging the gap between academic training and society and industry needs, particularly in the fields of mobility, health, and manufacturing.

## Methods

**Fig. 1:**
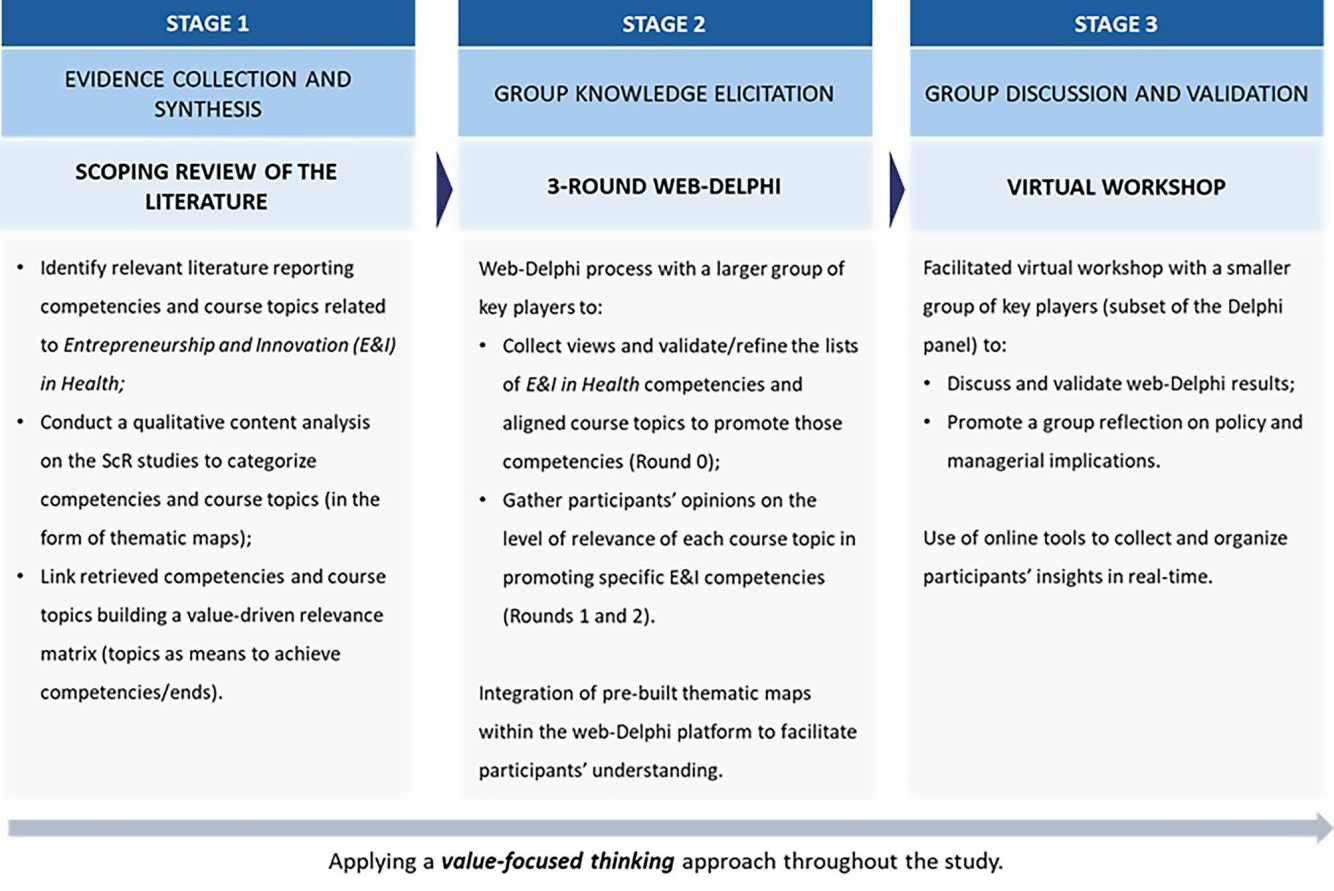
Overview of the methodological approach employed in this study

Figure 1 presents the 3-stage methodological approach adopted in this study, designed according to the principles of Collaborative Value Modelling (CVM) [45] and Value-Focused Thinking (VFT) [41]. The CVM is a socio-technical framework designed to enhance participatory decision-making in complex, multi-stakeholder contexts. CVM integrates two complementary processes: first, a participatory environment using Web-Delphi to engage a large and diverse group of stakeholders; second, a collaborative environment within a decision conferencing process with a smaller group of key players [45]. The VFT approach emphasizes the importance of identifying fundamental objectives before considering the alternatives on how to achieve them. VFT distinguishes between “ends” (*what we want to accomplish*) and “means” *(how we accomplish it*), ensuring that solutions are aligned with core objectives rather than being driven by available alternatives [46]. This combined theoretical foundation enables our research to systematically address two critical questions: “*What should be achieved?” (identifying* competencies through group reflection and agreement) and *“How to achieve it?”* (linking educational content to competency development). The CVM framework ensures we capture diverse stakeholder perspectives while building shared knowledge, while VFT provides the structure for systematically connecting competencies with educational curricular topics.

### Stage 1: scoping review

A scoping review (ScR) was designed to address two primary research questions: (1) *What insights can be derived from the literature regarding competencies in Entrepreneurship and Innovation (E&I) considered relevant for current and future health professionals*? and *(2) Which course topics/subjects related to E&I have been incorporated into higher education health programmes/courses?* The ScR was conducted in accordance with the methodological framework delineated by Arksey and O’Malley [47] and the PRISMA Extension for Scoping Reviews (PRISMA-ScR) [48] guidelines.

A comprehensive search of the literature was conducted on the 21st February 2024 on the following electronic databases: MEDLINE, Scopus, Web of Science, EBSCO and Google Scholar. A structured search strategy was developed and applied on the title, abstract and keyword fields combining relevant search terms such as ‘entrepreneurship’, ‘innovation’ AND ‘health*’ AND ‘education’, ‘course*’, ‘competencies’, ‘skills’ (and respective synonyms). The searches were limited to studies written in English with no temporal restrictions imposed (see full search strategy in Additional File 1).

The following inclusion criteria were set for this review: peer-reviewed journal articles or reviews wherein either (1) competencies associated with E&I in Health are reported or discussed, either resulting from prior literature or from the results of a qualitative enquiry (e.g. survey, focus group, Delphi panel, workshop, etc.) or (2) course topics associated to E&I in health are identified or discussed, under the scope of the design, planning, implementation or evaluation of an educational initiative (e.g. undergraduate or post-graduate programme, workshop, etc.) promoted or delivered by a higher education institution. Records were excluded if they did not meet the predefined inclusion criteria, if not focused on entrepreneurial and/or innovation education for present and future professionals working within the health sector, or if did not have a focus on the health sector. Records presented as abstracts, conference proceedings, working papers, books and book sections, case reports, posters, editorials, letters, notes, commentaries, grey literature or without available full were excluded.

All references identified through the searches were imported into the reference management software ENDNOTE X7, and duplicates were removed. An initial screening training set comprising 10% of the sample was assessed to ensure the appropriateness of exclusion criteria and their consistent interpretation. The screening process comprised two stages. First, titles and abstracts of the retrieved studies were screened by one author (EM) and analysed against the predetermined inclusion and exclusion criteria. Subsequently, full-text articles were obtained and thoroughly assessed for eligibility by two authors (EM and FC), and validated by a third author (MDO). Any conflicts which arose during the screening process were resolved by consensus among all the authors.

Data extraction was conducted by one author (EM) and subsequently validated by two co-authors (MDO and FC) using a data extraction form specifically designed for this specially for this review and constructed in Microsoft Excel. The data collected pertained to study publication details, including the names of the authors, year and journal of publication, article title, study objective, study location, research design and data collection methods, E&I competencies reported, E&I course topics reported (in instances of design/implementation/evaluation of an educational programme addressed by a higher education institution on E&I in health), and target audience (beneficiaries of the competencies and/or educational topics reported/discussed in the study). The results, stored in the form of a spreadsheet, formed the basis for our analysis.

To analyse and synthesize information on E&I competencies and topics reported in the included studies, we employed qualitative content analysis (QCA) [49, 50]. QCA is a systematic research method used to analyse and interpret the content of textual data; it involves a systematic process of subjective interpretation, coding, and identifying themes or patterns within large volumes of text [51]. QCA was particularly well-suited to our research objectives for several reasons. First, it enables the systematic synthesis of multifaceted concepts, such as competencies and educational topics, which are often described differently across diverse literature sources. Second, QCA’s emphasis on contextual meaning allows for the preservation of important nuances in how E&I competencies are conceptualized within different health professional contexts. Third, the method’s systematic coding procedures ensure transparency and reproducibility. The first author (EM) read and re-read all included studies to familiarise himself with the content, identifying all textual segments related to E&I competencies and course topics. Relevant textual content was coded using an open coding scheme, allowing codes to emerge directly from the data rather than being predetermined. Codes were developed iteratively, with new codes added as novel concepts emerged and existing codes refined as understanding deepened. Throughout this process, a second author (MDO) independently validated the coding scheme and category development to ensure analytical rigor and reduce potential bias. Codes were then systematically grouped to generate sub-categories and overarching categories, which were collaboratively reviewed and refined by both authors before final validation by all authors. The final step involved organizing the validated categories into coherent and distinct thematic maps: one for ‘E&I in health’ competencies and other for ‘E&I in health’ course topics.

In order to link these two sets, the aforementioned VFT [41] approach was employed. Departing from the information within thematic maps and guided by the question “*does teaching topic X provide a key contribution to developing a specific E&I domain of competency Y in a professional working in the health sector?”* we were able to design and fill in a value-driven E&I competencies-topics relevance matrix. Here, we define *key contribution* as: *‘a topic that is fundamental to the development of one or more competencies encompassed in a specific E&I domain of competency*. The matrix was completed by the first author (EM) and subsequently validated by the two co-authors (MDO and FC). It served as the input information for the initial round of the web-Delphi process.

### Stage 2: web-Delphi process

The Delphi is a structured, interactive group communication process that aims to systematically collect expert perspectives and achieve a group alignment on a specific topic or issue [52]. It involves multiple rounds of anonymous questionnaires, with controlled feedback ― whereby participants receive structured summaries of anonymized responses from previous rounds ― being incorporated after each round to refine opinions and converge on a collective agreement [53]. Over recent decades, Delphi processes have increasingly been conducted within web-based platforms (known as web-Delphi or e-Delphi) [54] facilitating the seamless participation and involvement of a large number of experts and other stakeholders, who can answer the questionnaire at their own pace without temporal or geographical constraints. Moreover, the user-friendly design of the platforms and the integration of features that assist in answering the questionnaires (e.g. help buttons) enhance data quality and allow participants to submit their responses automatically, thereby increasing participants’ satisfaction and subsequently mitigating data transcription errors [45].

We conducted a modified three-round web-based Delphi to collect experts’ opinions and perspectives on: (1) the lists of E&I competencies and course topics retrieved from the scoping review of the literature (2) on the relevance of each course topic as a means to promote a specific set of E&I competencies. In a modified Delphi process, pre-selected items can be derived from an evidence synthesis and are used as an input to inform the first round of a Delphi process [55].

### Participants selection and recruitment

Participants selection followed a purposive sampling strategy [56] focusing on potential employers working within the health sector and on experts in health entrepreneurship and health innovation. Experts within the following four groups were targeted: health industry (e.g. pharmaceutical, medical devices’ and digital health companies, start-ups and health business incubators), health care providers (public, private and social sector institutions), government/public health administration institutions, and academia. Specifically, selected individuals should: (a) have professional knowledge and experience working within the health sector, including experience in human resources recruitment tasks; (b) have a specific interest in the field of E&I in health; (c) be potentially available to participate in all Delphi rounds given the timeline planned; (d) not have any conflict of interest preventing their impartial participation.

### Delphi Preparation

**Fig. 2:**
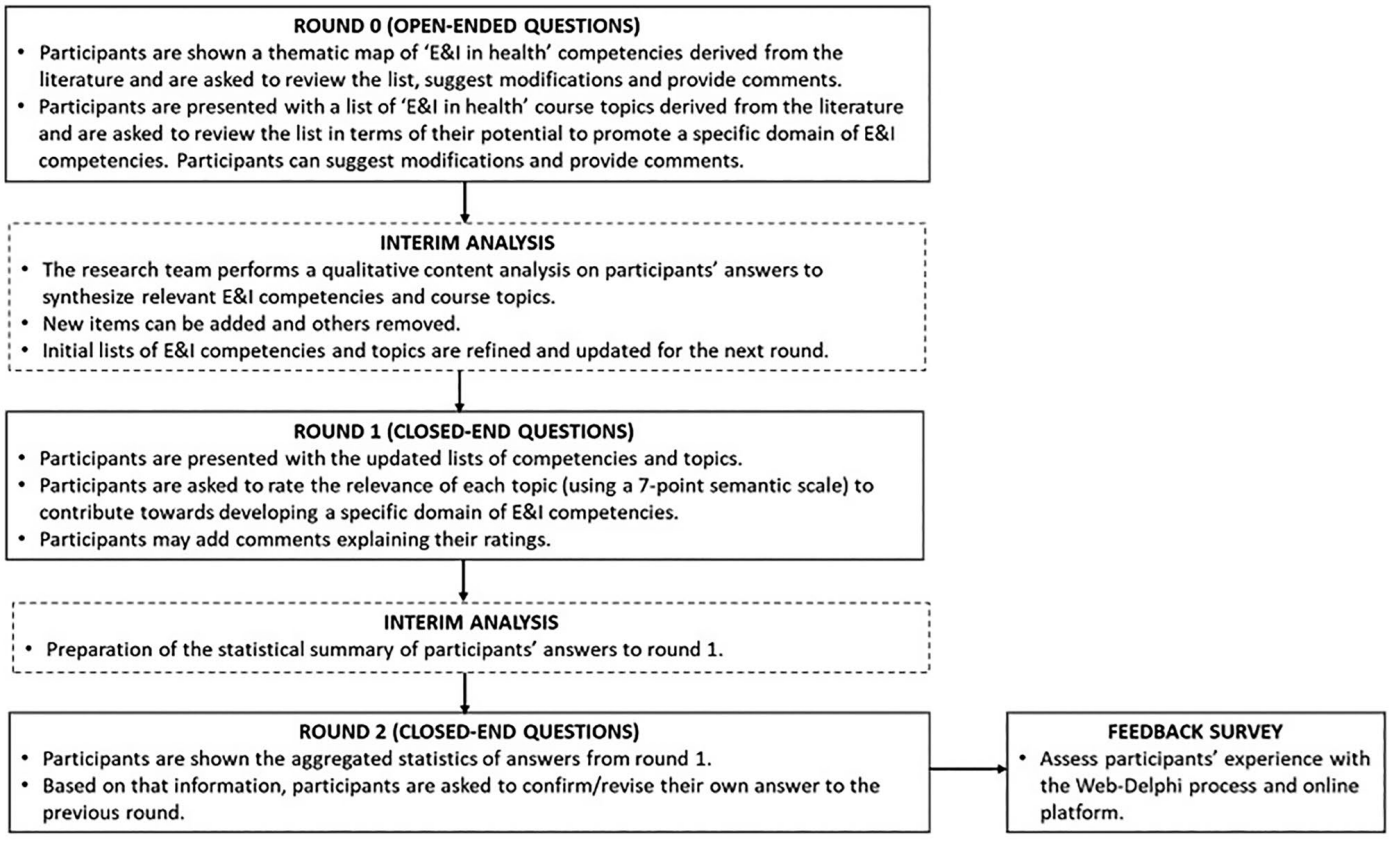
Flowchart depicting the 3-round web-Delphi process

The Delphi process was implemented in the Welphi [57] online platform and was composed of three rounds (see Fig. 2). Before each round, an e-mail was sent to each participant delineating the scope and objectives of the respective round, the timeline of the Delphi process, and instructions for accessing and operating within the Welphi platform. Informed consent was obtained from all participants before starting the Delphi process. Participants were given a two-week period to respond to each round. A maximum of three e-mail reminders were sent to non-respondents during each round.

### The 3 rounds of the web-Delphi process

**Fig. 3:**
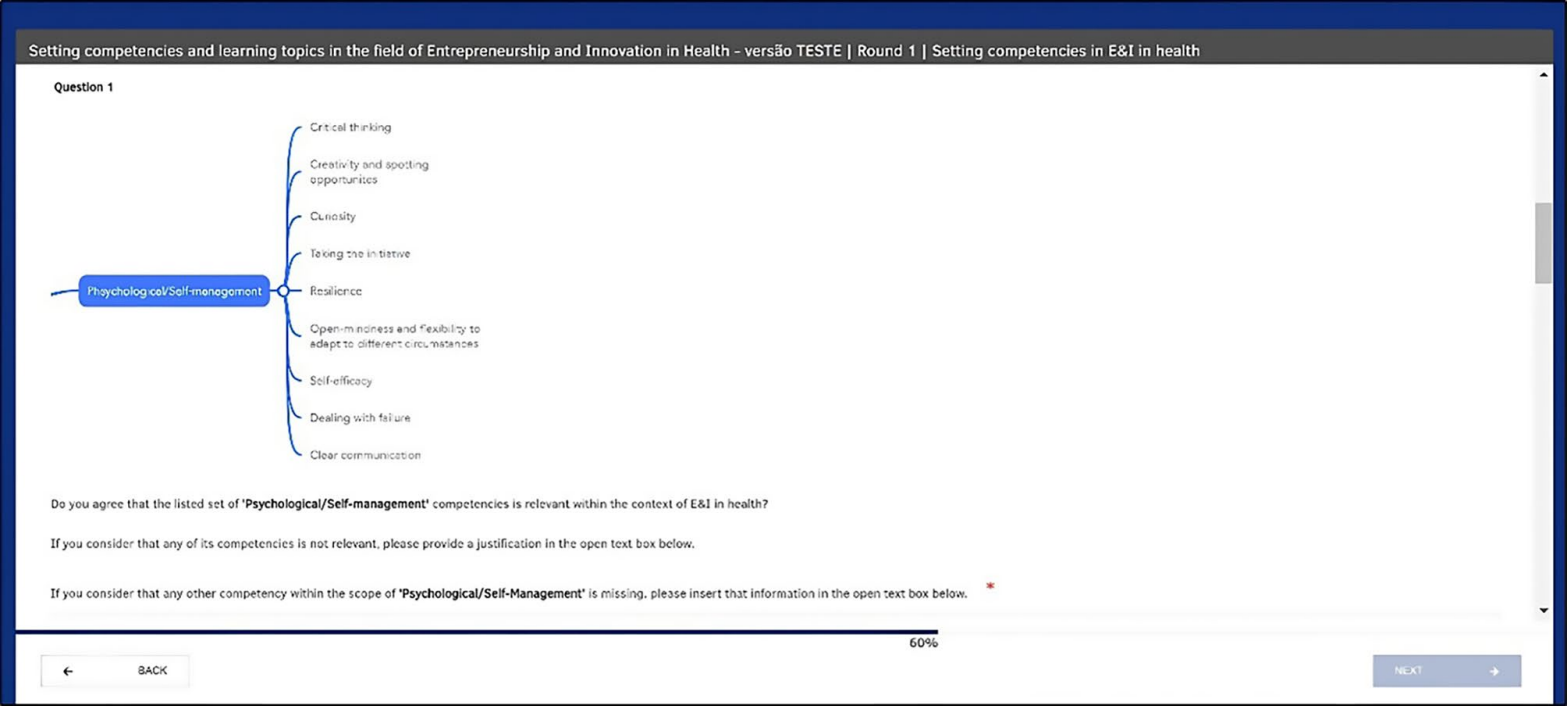
Illustration of web-Delphi Round 0 questionnaire screen (example for ‘Psychological/Self-management’ E&I domain of competencies)

For Round 0, two open-ended questionnaires were designed. In the first questionnaire, participants were presented with initial list of *E&I in health* competencies (grouped in the five specific E&I domains of competency) obtained from the ScR, and asked to review and comment the appropriateness of each competency presented within the context of E&I in health. Participants were invited to suggest modifications (potential additions or deletions of items) and provide the respective justifications (see Fig. 3). The second questionnaire pertained to the list of E&I course topics derived from the ScR. However, in this instance, and in an attempt to guide the participants towards a VFT framing, we presented the course topics grouped within the respective E&I domain of competency (based on the output from the value-driven competencies-topics relevance matrix completed during the ScR stage). Again, participants were given the opportunity to review and comment on each item of the list, suggest modifications (potential additions or deletions of items) and provide the respective justifications.

Upon completion of Round 0, participants’ responses were analysed and synthesized using again a QCA approach. The initial lists of competencies and topics were updated - taking into account the analysis of participants’ inputs - and presented in the subsequent round. Round 0 was conducted between 24 May 2024 and 13 June 2024.

**Fig. 4:**
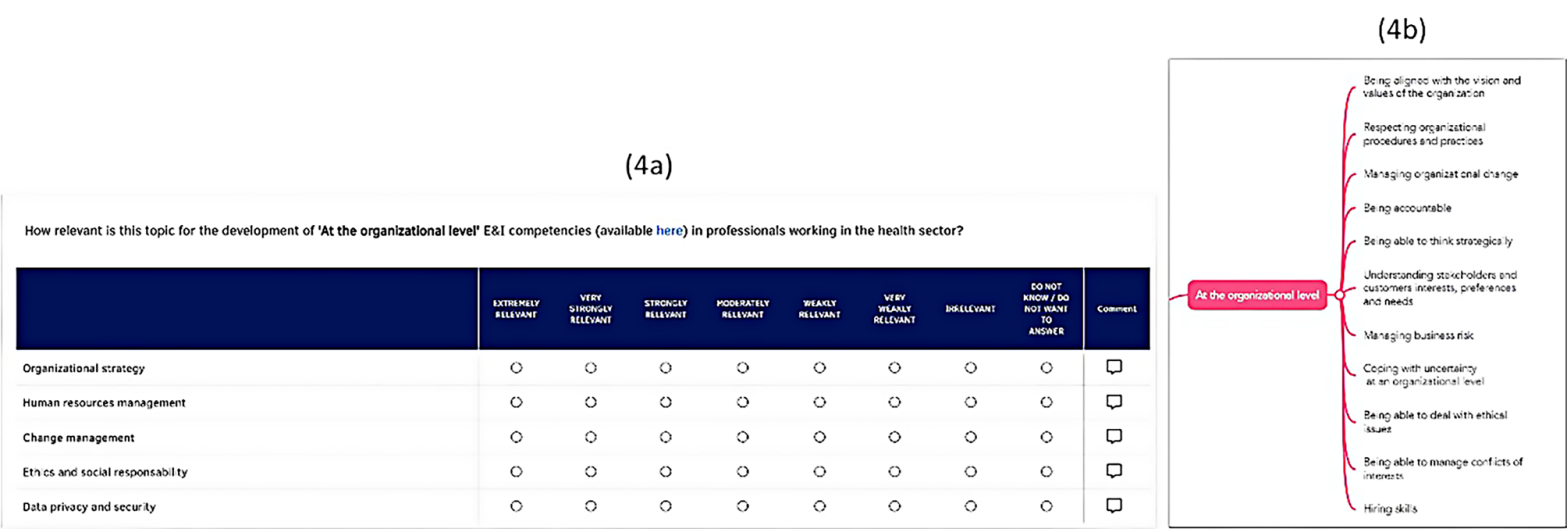
Illustration of the Round 1 questionnaire screen. (**a**) Participants were asked to rate the relevance of each topic in contributing to the development of E&I competencies (example for ‘At the organizational level’ E&I competencies). (**b**) By clicking on the hyperlink ‘Available here’, participants could access all the ‘At the organizational level’ E&I competencies (presented as a subset of the refined ‘E&I in health’ competencies thematic map)

For Round 1, one closed-end questionnaire was created. Participants were invited to give their opinion about the relevance of each course topic as a means to develop a specific E&I domain of competency. Before answering the questions, participants were presented with a visual representation of a thematic map (embedded within the web-Delphi questionnaire) depicting each E&I domain of competency and its respective competencies. Participants could choose one level of the 7 only one category of the following 7-point relevance (semantic) scale: ‘Extremely relevant’, ‘Very Strongly relevant’, ‘Strongly relevant’, ‘Moderately relevant’, ‘Weakly relevant’, ‘Very weakly relevant’ and ‘Irrelevant’ (scale adapted from [58]). Moreover, for each course topic participants could select the ‘Don’t know/Don’t want to answer’ option or provide specific comments (see Fig. 4). Round 1 was conducted between 14 June 2024 and 28 June 2024.

In Round 2, participants had access to similar questionnaire screens displayed in Round 1 (see Additional File 2). Yet, they could access their own previous answers together with a summary of the distribution of all participants’ responses to Round 1 (that could be seen either in percentage or numbers) and all comments provided in the previous round. In light of that information, participants could modify or maintain their previous answers. Round 2 occurred within 5 July 2024 and 19 July 2024.

Upon completion of the web-Delphi process, participants were invited to respond to a brief feedback survey regarding their overall experience in participating in this web-Delphi (see Additional File 2). The survey comprised three closed-ended questions to assess participants’ level of agreement with the Delphi results and an open-ended question wherein participants could provide comments and suggestions. It should be noted that the completion of this feedback survey was not mandatory.

### Analysis of results: agreement on topic relevance and opinion change

To synthesise and analyse participant responses from the Delphi process, we conducted analyses at multiple levels. Primary analyses examined voting patterns at the overall panel level, incorporating responses from all 29 participants. Secondary analyses explored variations in perspectives between the different stakeholder groups. These comparative analyses were conducted only when sample sizes were sufficient to ensure meaningful interpretation of findings.

A group majority opinion was determined for each topic using the following approach: when a single relevance category received more than 50% of responses, it was designated as the group majority opinion. When no individual category exceeded 50%, several consecutive relevance categories surpassing 50% were considered as the group majority opinion (in cases of draws, consecutive categories with lower relevance were taken).

Then, to assess agreement on topic relevance, we considered the percentage of responses in the three highest relevance categories (Extremely Relevant, Very Strongly Relevant, and Strongly Relevant) as the agreement metric, and an agreement threshold of 70% was assumed as the minimal level for considering that the panel deems the topic as unquestionably relevant (meaning that at least 70% of participants considered the topic to be at least strongly relevant). This agreement threshold is consistent with other Delphi studies [59].

Moreover, following good practice in Delphi research, opinion change was assessed: changes in the percentage distribution of relevance votes, between Round 1 and Round 2, were observed for each course topic; additionally we calculated the rate of opinion change for each stakeholder group and for the overall panel, defined as the number of actual opinion changes divided by the total number of possible opinion changes (i.e., number of participants × number of topics), following the approach used in [60].

### Stage 3: virtual workshop

A virtual workshop was conducted to present web-Delphi results and prompt a discussion on higher education implications that could be derived from the study. Also, and taking stock of experts’ knowledge of the realities of the Portuguese education ecosystem, particularly within the context of University of Lisbon (ULisboa), we aimed to gather their perspectives about how ULisboa can – taking stock of the results of this study – leverage the opportunity of promoting E&I courses and training for current and future professionals working in the health sector.

The criteria for selecting potential candidates to participate in the workshop were as follows: (1) participation in the previous stage of this study (the 3-round Delphi process) and (2a) involvement in past health-related initiatives or projects with ULisboa, or (2b) working within ULisboa in the area of entrepreneurship.

To ensure flexibility given the participants’ time and travelling constraints, the workshop was conducted in a virtual format. Zoom© software was utilised as the videoconferencing tool, and MIRO© web-based whiteboard platform was employed to gather and categorise participants’ views in real-time. A MIRO© board template specifically designed for the workshop activities was developed by the facilitation team and evaluated prior to implementation.

The facilitation team comprised four individuals: two individuals responsible for conducting the workshop (MDO and FC), engaging with the group and managing the group discussion, and two other individuals responsible for documenting notes in MIRO© software and categorising participants’ perspectives and insights (EM and another colleague). One member of the University of Lisbon board of directors attended as an independent observer.

The virtual workshop occurred on 17 October 2024 and had a duration of 1 h and 15 min. Prior to the start of the workshop, informed consent was obtained from all participants for the recording of the virtual session (for notetaking and verification purposes).

The initial segment of the workshop comprised a brief overview of the study’s methodological approach, followed by a presentation of the key findings from the 3-round web-Delphi process. Subsequently, a group discussion took place around two primary questions: *“(1) Which insights and implications do you take from the Delphi process?”* and *“(2) Which opportunities do these results bring to ULisboa regarding E&I courses and training for current and future professionals working in the health sector?* Prior to the workshop, participants received information on these questions and also a report with the results of the Delphi process.

## Results

### Scoping review results

**Fig. 5:**
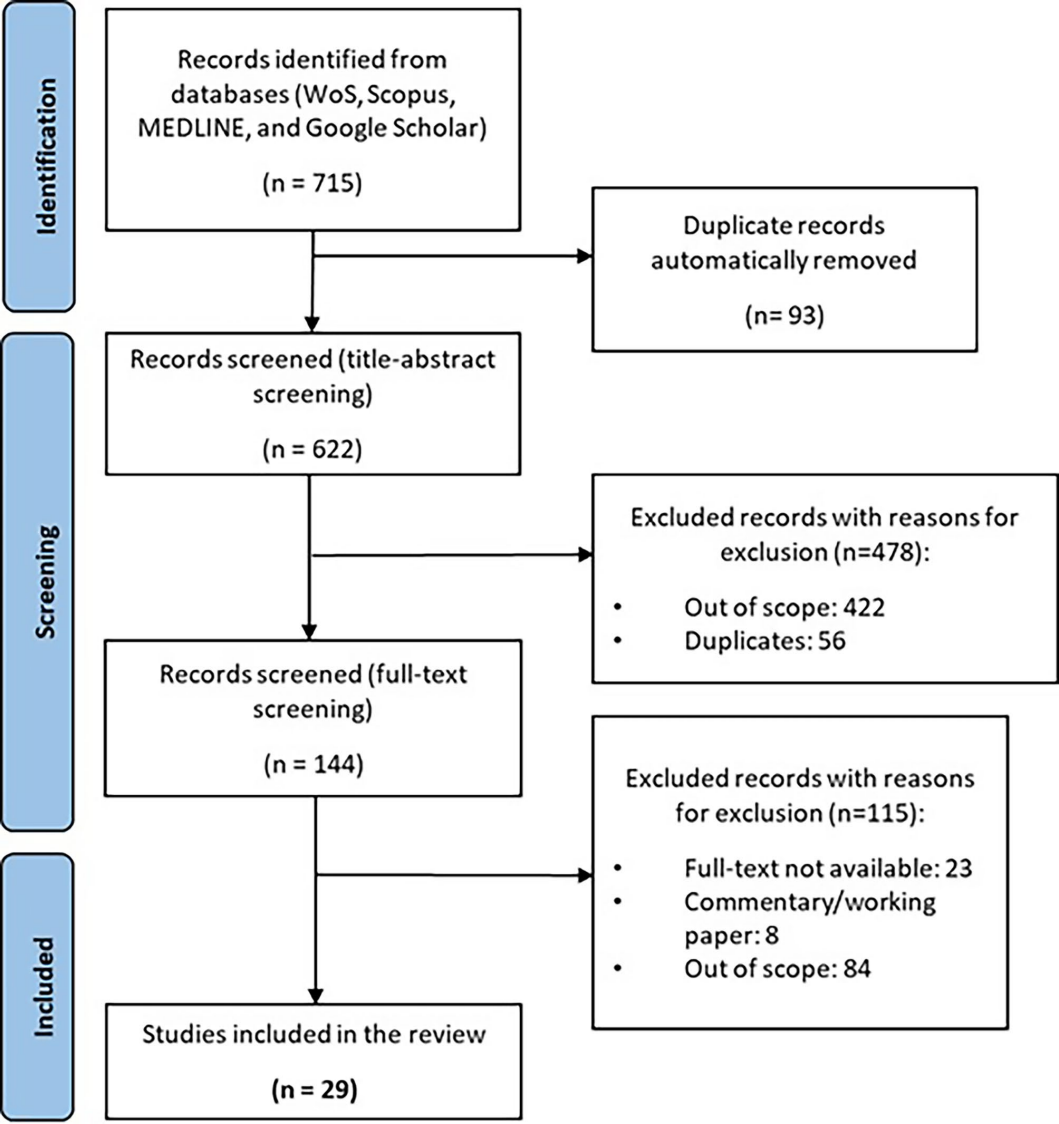
PRISMA flowchart illustrating the selection of studies included in the scoping review

The structured search process yielded 715 studies, which were subsequently reduced to 622 following the automatic removal of duplicates. Upon screening the titles and abstracts, 478 studies were excluded, resulting in 144 studies for full-text assessment. Of these, 115 were excluded due to being out of scope (*N* = 84), having inaccessible full text (*N* = 23), or being classified as commentaries, notes, editorials, or working papers (*N* = 8). In the end, 29 studies met the inclusion criteria and were included in the scoping review (see Fig. 5).

The 29 studies included in this scoping review span from 2005 to 2024, with a notable increase in publications in from 2019 onwards (accounting for 69% of the studies). Considering the country of affiliation of the first author, the USA accounts for the majority of studies (*N* = 11, 37.9%), followed by the United Kingdom (*N* = 4, 13.8%). Other countries, including Lebanon (*N* = 2, 6.9%), and Brazil, Saudi Arabia, Israel, China, New Zealand, Spain, Canada, Ireland, Costa Rica, Iran, Finland, and Finland, with one study each. This global distribution highlights the widespread recognition of the importance of fostering entrepreneurial competencies and E&I educational frameworks within health education programs.

The studies reviewed employed a range of research designs, reflecting diverse methodological approaches. Qualitative designs were the most prevalent, used in 9 out of 29 studies (31.0%), primarily using semi-structured interviews and document analyses. Quantitative approaches were applied in 7 studies (24.1%), often utilizing cross-sectional surveys. Mixed methods were employed in 5 studies (17.2%), combining approaches such as Delphi panels, interviews, reviews, among others. Descriptive program descriptions or evaluations were found in 6 studies (20.7%). Two studies (6.9%) were literature reviews, including one systematic review and one scoping review.

The target audience of the reviewed studies varied, with healthcare professionals such as physicians, nurses, pharmacists, and health managers being the primary focus in 15 out of 29 studies (51.7%). Health sciences students, including undergraduate, graduate, and postdoctoral trainees, were targeted in 14 studies (48.3%).

It was possible to categorise retrieved studies into two distinct groups, henceforth referred to as Group A and Group B. Group A comprises studies that focus on the E&I competencies/skills that are relevant for health sector professionals in the present and future (*N* = 16). Conversely, Group B consists of studies that report on E&I course topics/subjects which have been addressed and incorporated into university health education curricula (*N* = 13). It is noteworthy that none of the studies reviewed discussed or reported on both E&I competencies and topics in an integrated and concomitant manner. The complete list and detailed characterization of ScR studies can be found in Additional File 1.

**Fig. 6:**
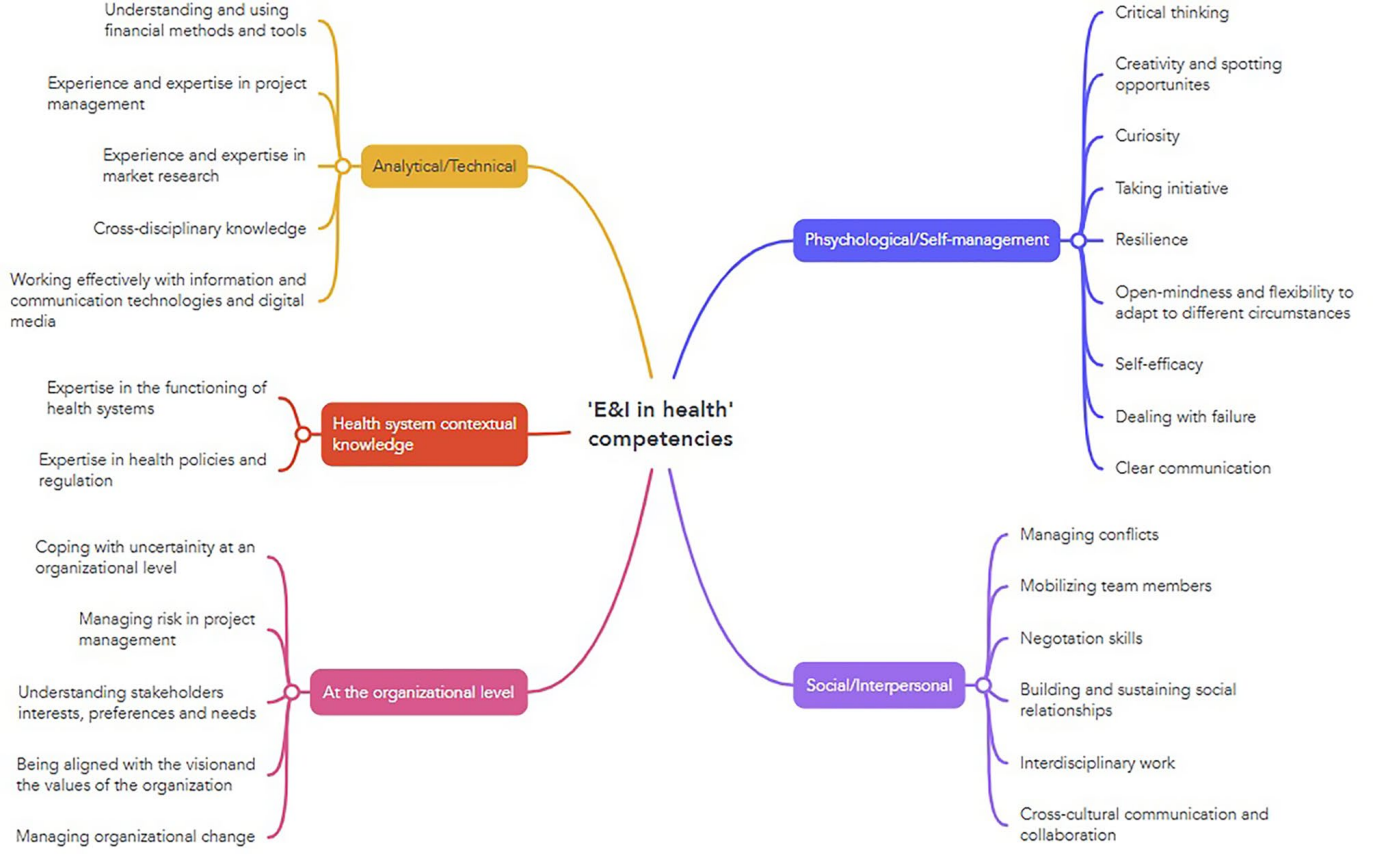
‘E&I in health’ competencies thematic map (from qualitative content analysis on scoping review studies)

Following the QCA conducted on Group A studies, it was possible to generate a thematic map of ‘E&I in health’ competencies, comprising 28 distinct items which were further grouped in the following five E&I domains of competency: Psychological/Self-management’, ‘Social/Interpersonal’, ‘At the organizational level’, ‘Analytical/technical’ and ‘Health system contextual knowledge (see Fig. 6). ‘Psychological/Self-management’ concern an individual’s personality, attitudes, and perceptual-cognitive abilities; Social/Interpersonal’ competencies refer to the skills and abilities that enable an individual to effectively interact, communicate, and build relationships with others. ‘At the organizational level’ competencies mainly refer skills and attitudes an individual must possess to operate effectively within an organization and align with its goals and culture. ‘Analytical/Technical competencies’ refer to specific skills and knowledge required to perform particular tasks or use certain analytical methods, technologies and tools. ‘Health System contextual knowledge’ refers mainly to the specific understanding required to navigate and contribute to the improvement of health systems.

Following the QCA conducted on Group B studies, it was possible to create a list of E&I in health course topics, that includes 34 distinct items, which were subsequently organized in in five distinct Topic Areas: ‘*Psychology in Business and Economics’*,* ‘Management and Leadership’*,* ‘Health systems and policies’*,* ‘From idea to market’ and ‘Ethics’.*

**Fig. 7:**
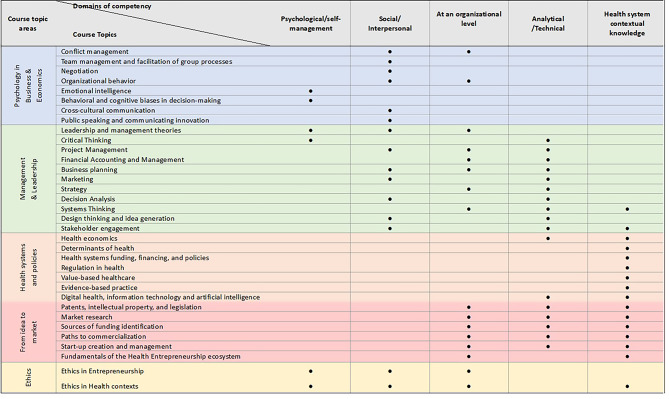
‘E&I in health’ competencies-topics relevance matrix

Figure 7 illustrates the ‘E&I in health’ value-driven competencies-topics relevance matrix, designed to serve in health entrepreneurship. A marked cell indicates that the corresponding topic is fundamental to the development of one or more competencies encompassed in a specific E&I domain of competency. As opposed, an empty middle cell indicates that the topic does not contribute to develop the respective domain competencies. The matrix served as input information for the initial Delphi initial round.

### Web-Delphi results

*N* = 53 individuals were identified and were formally invited via e-mail, of which *N* = 29 agreed to participate in the Delphi process. The participants were recruited during the first two weeks of May 2024. The 29 participants participated in all rounds, there being a 0-dropout rate in successive rounds. The self-reported demographic information of the participants is presented in Table 1.

**Table 1:** Demographic information of the 29 individuals who participated in the web-Delphi process

Demographic information	*N* = 29	%
**Gender**		
Male	15	52%
Female	14	48%
**Affiliation**		
Health industry	13	45%
Health care providers	8	28%
Academia	7	24%
Government	1	3%

### Validation and refinement of E&I competencies and aligned course topics (Round 0)

Concerning Round 0, participants actively proposed additions and revisions to the initial lists of E&I competencies and course topics derived from the literature: 24 additional competencies were identified and added to the initial list of five E&I domains of competency that emerged from the literature (no new domains emerged in Round 0). Furthermore, 23 new course topics added to the original list of course topics, as detailed in Table 2.

**Table 2:** List of new competencies and new course topics (per E&I domain of competency) that emerged from the analysis of participants answers to round 0

Domain of competency	New competencies	New course topics
*Psychology/Self-management*	‒ Empathy and emotional intelligence ‒ Ability to build trust ‒ Self-confidence and self-motivation ‒ Being humble ‒ Engaging in lifelong learning ‒ Entrepreneurial mindset ‒ Good life-work balance	‒ Know Thyself (Psychology of Personality ‒ Mental health and well being ‒ Professional Resilience ‒ Risk Taking and dealing with failure ‒ Time and stress management
*Social/Interpersonal*	‒ Leadership skills ‒ Teamworking skills ‒ Openness and willingness to give and receive feedback ‒ Recognizing talent	‒ Media training
*At the organizational level*	‒ Respecting organizational procedures and practices ‒ Hiring skills ‒ Being accountable ‒ Being able to think strategically ‒ Being able to deal with ethical issues ‒ Being able to manage conflicts of interests	‒ Human resources management ‒ Change management ‒ Ethics and social responsibility ‒ Data privacy and security
*Analytical/Technical*	‒ Experience and expertise in marketing ‒ Experience and expertise in business/management models ‒ Experience and expertise in quality and risk management systems	‒ Risk management ‒ Quality Management ‒ Lean Management ‒ Data analytics, big data and artificial intelligence
*Health system contextual knowledge*	‒ Knowledge in evidence-based medicine and health technology assessment of distinct technologies ‒ Knowledge in health economics and policy ‒ Knowledge in health regulations and legal frameworks ‒ Knowledge in health organizations’ management	‒ Pharmacoeconomics and Health Technology Assessment ‒ Health geopolitics and public affairs ‒ Regulation in health ‒ Regulation and legal frameworks in digital health ‒ Hospital management ‒ Open innovation ‒ Sustainability and innovation management ‒ Business case analysis ‒ Digital health, information technology and information technology systems interoperability and integration

The final list of E&I competencies comprises 51 distinct items: 16 Psychological/Interpersonal competencies, 10 Social/Interpersonal competencies, 11 ‘At the organizational level’ competencies, 9 ‘Analytical/technical competencies’ and 5 ‘Health system contextual knowledge’ competencies). Moreover, the final list of course topics includes 55 different items: 8 topics associated with the development of Psychological/self-management competencies, 9 topics associated with associated with the development of Social/Interpersonal competencies, 5 topics associated with the development of ‘At the organizational level’ competencies, 11 topics related to development of ‘Analytical/Technical’ competencies and lastly 22 topics related to the development of ‘Health system contextual knowledge’ competencies. The refined thematic maps that include the final list ‘E&I in health’ competencies and ‘E&I in health’ course topics can be found in Additional File 2.

### Relevance of each course topic to promote E&I competencies

Table 3 presents results from the final round (Round 2), displaying the percentage distribution of relevance ratings for all 55 course topics organized by their corresponding E&I domains of competency. For each topic, the table indicates the panel majority opinion (applying the rules described in Methods, Stage 2 sub-section) and specifies whether agreement on topic relevance was achieved according to the predetermined 70% threshold.

**Table 3:**
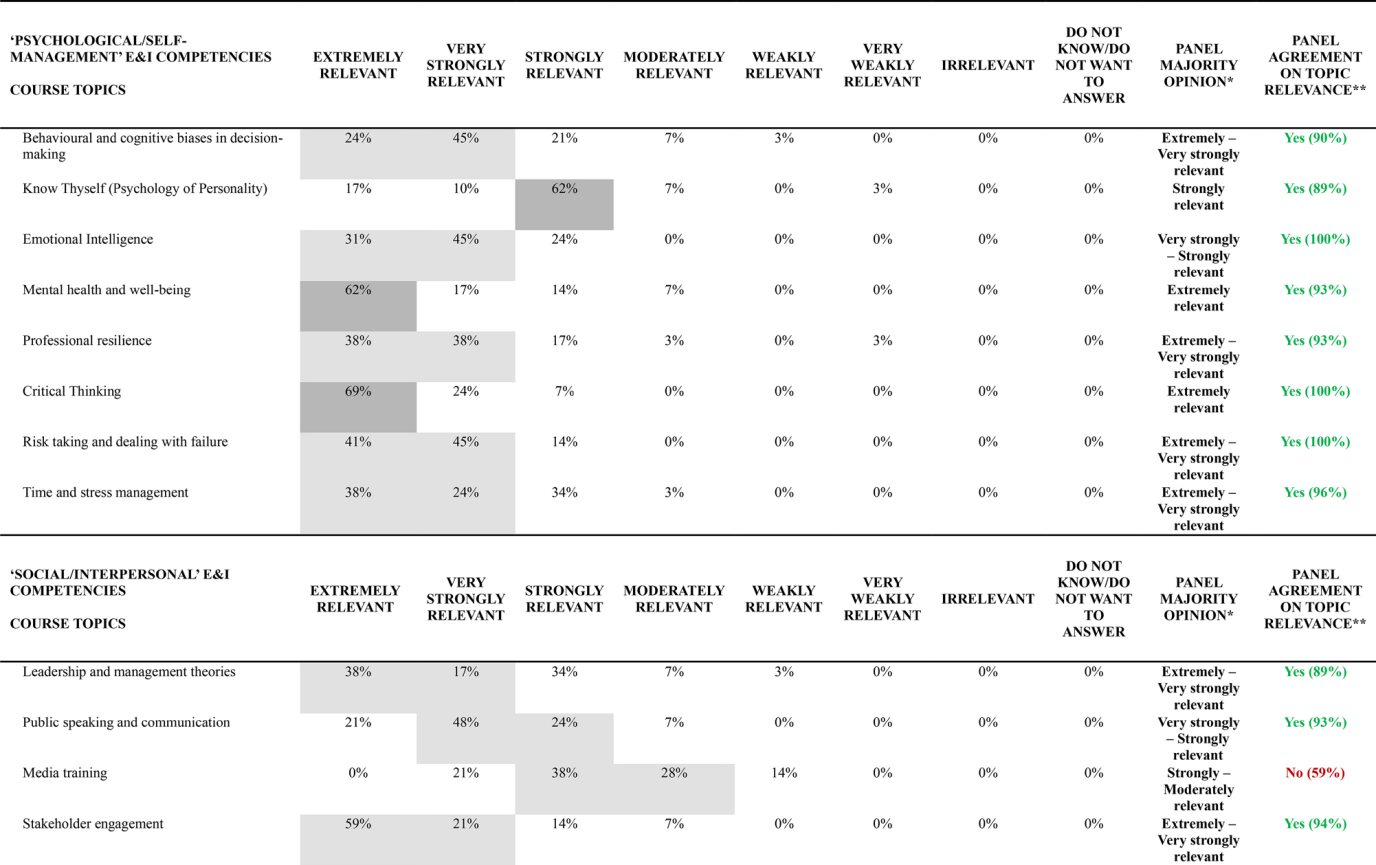

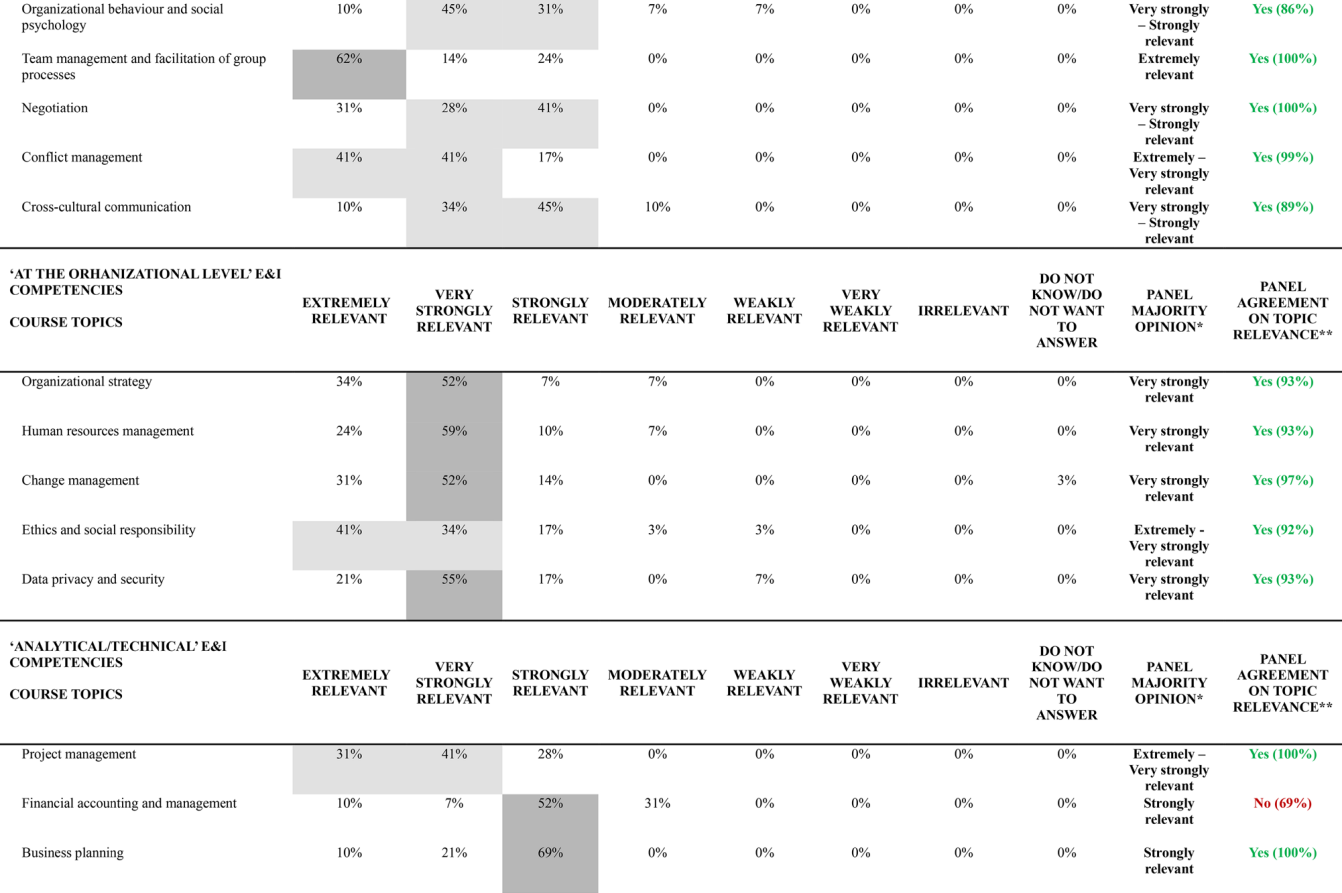

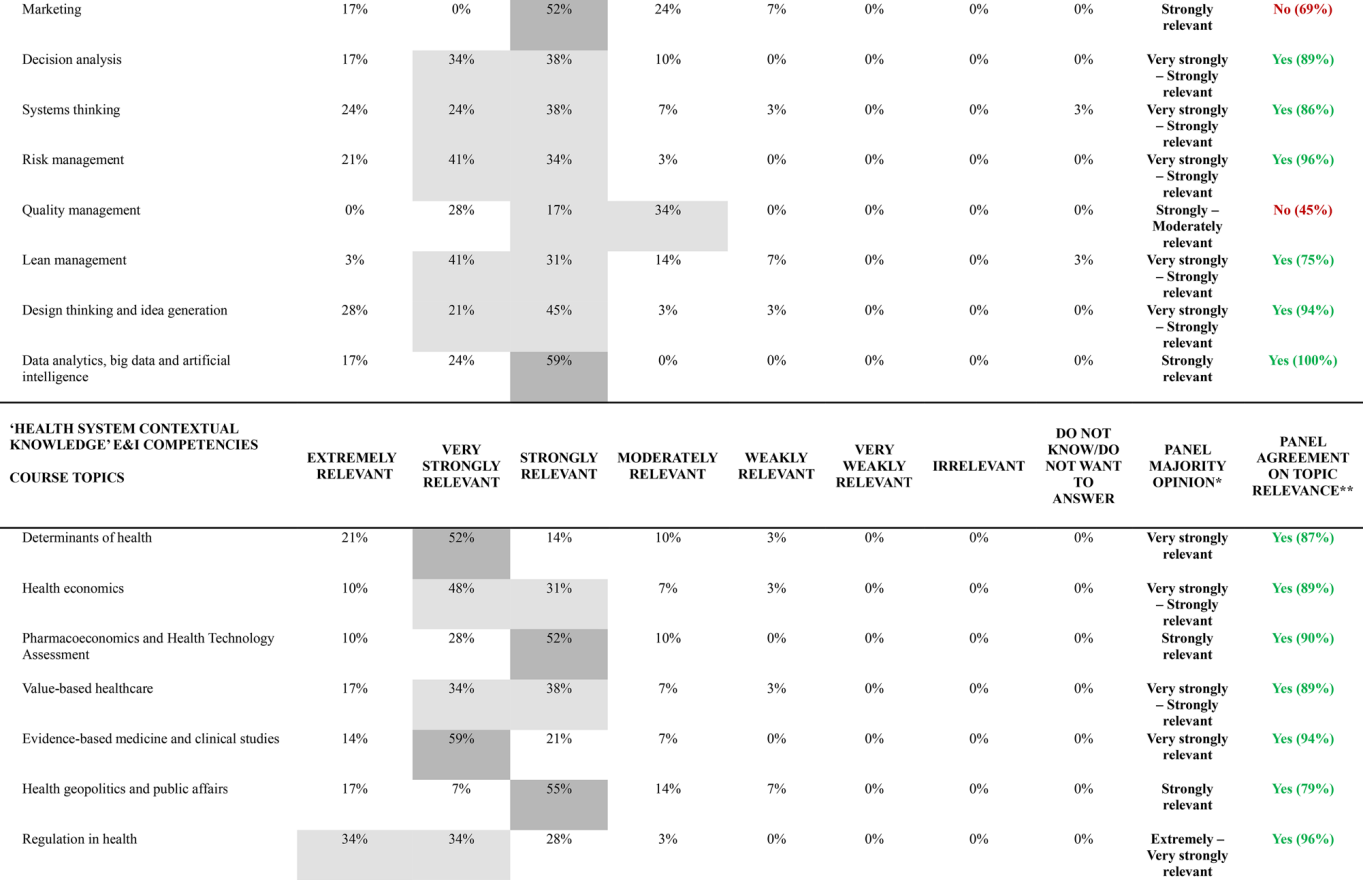

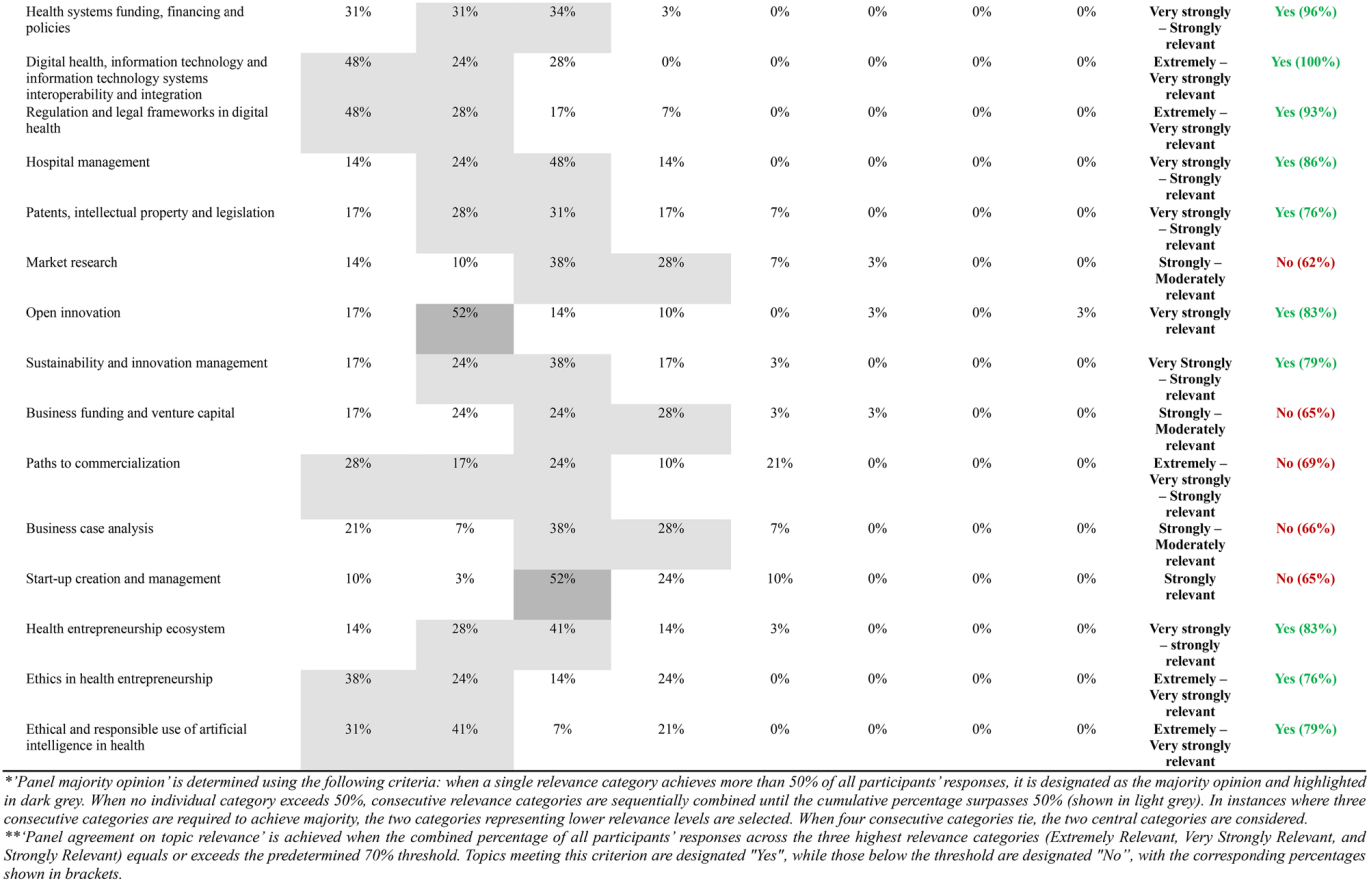
Web-Delphi round 2 distribution of results concerning the relevance of each course topic to promote a specific E&I domain of competency

The majority opinion analysis reveals distinct patterns across the 55 course topics, but with a majority of topics − 50 (91%) – gathering a majority opinion of at a least ‘Strongly relevant’. Three topics gathered an ‘Extremely relevant’ group majority opinion, 16 topics an ‘Extremely to Very strongly relevant’ group majority opinion, 7 topics a ‘Very Strongly relevant’ majority, 16 a ‘Very Strongly’ to ‘Strongly relevant’ majority, and 8 a ‘Strongly relevant’ majority. Of note, no course topics gathered a majority on ‘Irrelevant’, and not even on the categories ‘Weakly relevant’ and ‘Very weakly relevant’.

Of the 55 course topics, 46 (84%) achieved the 70% relevance agreement threshold, with the remaining 9 (16%) falling below this criterion. Agreement patterns varied significantly across E&I domains of competency, with ‘Psychological/Self-management’ and ‘At the organizational level’ domains achieving complete agreement on the topics relevance (100%, 8/8 and 5/5 topics above the agreement threshold, respectively), followed by ‘Social/Interpersonal’ (89%, 8/9 topics) and ‘Health System Contextual Knowledge’ (82%, 18/22 topics) domains. The ‘Analytical/Technical’ E&I domain of competency demonstrated in general less agreement concerning the relevance of a larger proportion of topics, with only seven out of eleven topics (64%) reaching the agreement threshold. Table 4 presents the top 3 topics by E&I domain of competency (with highest percentage of agreement on topic relevance).

**Table 4:** Top topics by E&I domain of competency according to level of agreement (*N* = 29 participants)

Ranking	Psychological/self-management E&I domain of competency	Social/interpersonal E&I domain of competency	‘At the organizational level’ E&I domain of competency	Analytical/technical E&I domain of competency	Health system contextual knowledge E&I domain of competency
**#1**	Critical Thinking (100%)	Team management and facilitation of group processes (100%)	Change management (97%)	Project management (100%)	Digital health, information technology and information technology systems interoperability and integration (100%)
**#2**	Risk of dealing with failure (100%)	Negotiation (100%)	Organizational strategy (93%)	Data analytics, Big Data and AI (100%)	Regulation in health (96%)
**#3**	Emotional Intelligence (100%)	Conflict management (99%)	Human resources management (93%)	Business planning (100%)	Health systems funding, financing and policies (96%)

Topics were ranked based on the ‘Agreement on topic relevance’ metric, defined as ≥ 70% combined responses of ‘Extremely relevant,’ ‘Very relevant,’ and ‘Strongly relevant.’ The ranking hierarchy applied the following tie-breaking criteria: (1) highest ‘Panel Agreement on High Relevance’ percentage, (2) highest combined ‘Extremely relevant’ and ‘Very relevant’ percentage, and (3) highest ‘Extremely relevant’ percentage only. ‘‘Panel Agreement on High Relevance’ percentages are shown in brackets

Round 1 results can be accessed in Additional File 2. Of note, participants did not provide any comments during both Round 1 and Round 2.

### Stakeholder group analyses: agreement on topic relevance and opinion change

Detailed stakeholder group analyses can be accessed in Additional File 2. The Government group (*n* = 1) was excluded from group comparisons due to insufficient sample size.

Agreement rates varied substantially across the three stakeholder groups analysed (health industry n = 13 respondents, health care providers n = 8, academia n = 7). The health industry group demonstrated a higher agreement (above the 70% threshold) in a larger number of topics 53 out of 55 topics (96%). Only two topics failed to reach agreement: ‘Media training’ (62%) and ‘Business funding and venture capital’ (69%).

For participants from Academia, 48 out of 55 topics (87%) achieved agreement on topic relevance. Seven topics (all from ‘Health contextual knowledge’ E&I competency domain) failed to meet the threshold, namely ‘Market research’ (57%), ‘Open innovation’ (57%), ‘Business funding and venture capital’ (57%), ‘Paths to commercialization’ (57%), ‘Business case analysis’ (43%), and ‘Start-up creation and management’ (57%).

For participants from health care providers, 46 out of 55 topics (83%) meet the agreement threshold. Topics which did not reach the threshold included ‘Media training’ (38%), ‘Financial accounting and management’ (63%), ‘Marketing’ (63%), ‘Quality management’ (50%), ‘Patents, intellectual property and legislation’ (50%), ‘Market research’ (38%), ‘Sustainability and innovation management’ (63%), ‘Business funding and venture capital’ (63%), ‘Paths to commercialization’ (50%), ‘Business case analysis’ (38%), ‘Start-up creation and management’ (50%), and ‘Ethics in health entrepreneurship’ (63%).

Stakeholder group analyses revealed distinct agreement patterns across the distinct E&I competency domains. Small differences in opinion were observed within topics of the ‘Psychological/Self-management’ and ‘Social/Interpersonal’ domains of competency, for which near complete agreement on topic relevance was achieved across topics. Conversely, larger divergences were observed in the ‘Analytical/Technical’ and ‘Health System Contextual Knowledge’ domains. For instance, health industry participants consistently rated traditional business topics (e.g. marketing, financial management) as more relevant than healthcare providers and academia participant. In contrast, healthcare providers rated clinical and system-focused topics as more relevant compared to the other stakeholder groups.

Regarding opinion change, between Delphi Rounds 1 and 2, 220 opinion changes occurred out of 1595 possible changes across all participants answers to all topics, yielding an overall opinion change rate of 14%. Opinion stability varied by stakeholder group: Academia demonstrated the highest rate of opinion change at 18%, while health industry and health care providers both exhibited 12% of opinion change (see Additional File 2).

All topics had at least one opinion change associated. Topics with the highest observed opinion change included ‘Leadership and management theories’, ‘Organizational strategy’, ‘Organizational behaviour and social psychology’, and ‘Start-up creation and management’, with 7 changes each (out of 29). Conversely, topics demonstrating less opinion change included ‘Health systems funding, financing and policies’, ‘Negotiation’, ‘Business funding and venture capital’ and ‘Paths to commercialization’, with one change each.

### Delphi feedback survey results

All the 29 Delphi participants accessed the feedback survey administered in the final screen of Round 2. The results of the feedback survey can be found in Additional File 2, demonstrating high participant satisfaction with the overall Delphi process. 93% (27/29) either strongly agreeing or agreeing that they clearly understood the required tasks and received clear information from the facilitation team; 86% (24/28) either agreed or strongly agreed that the platform design was helpful for understanding tasks and sharing their views; and 78% (21/27) considered the aggregated statistics feature valuable for reflecting on their previous responses. Notably, none of the participants answered the open-ended question requesting additional comments or suggestions about their overall experience with this Delphi process.

### Workshop results

**Fig. 8:**
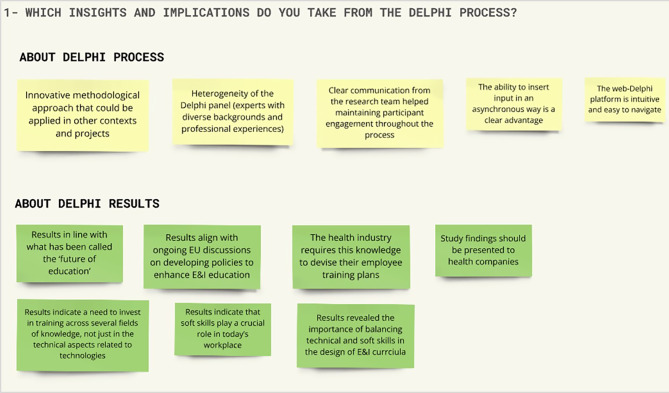
MIRO board synthesizing participants’ responses to the workshop’s first question. Workshop’s first question: **“**Which insights and implications do you take from the Delphi process?”

**Fig. 9:**
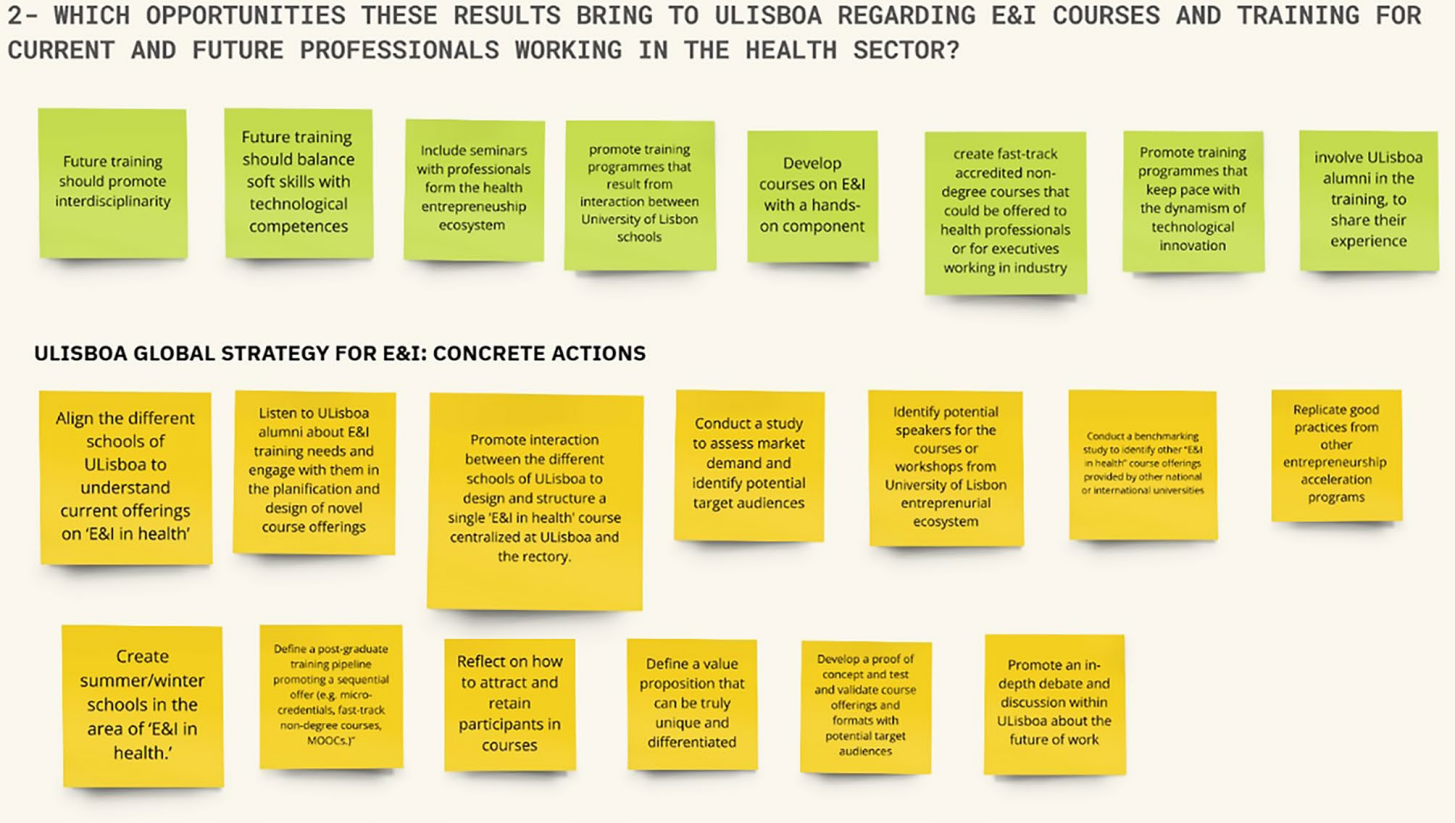
MIRO board presenting a synthesis of participants’ responses to the workshop’s second question. Workshop’s second question: “Which opportunities these results bring to ULisboa regarding E&I courses and training for current and future professionals working in the health sector?”

Of the nine experts who were formally invited to participate in the workshop, six experts accepted the invitation and participated in the workshop (one from academia, four from the health industry, and one from healthcare providers’ group). The six participants engaged actively in the discourse and provided their perspectives in response to the questions posed by the facilitation team. Participants’ contributions were categorised using differently coloured adhesive notes. Following the workshop, MIRO boards were distributed to workshop participants for further refinement and validation (see Figs. 8 and 9).

Regarding the question “*Which insights and implications do you take from the Delphi process?”* (full results synthesised in Fig. 8), participants emphasized the importance of engaging with a diverse and heterogeneous Delphi panel (with different academic and professional backgrounds), the innovation of the methodology applied and intuitive use of the Welphi platform. Furthermore, participants mentioned that Delphi results are in line with has been discussed and debated in the scope of EU.

Regarding the question “*Which opportunities do these results bring to ULisboa regarding E&I courses and training for current and future professionals working in the health sector?”* (full results synthesised in Fig. 9), participants provided several suggestions including: the design of interdisciplinary ‘E&I in health’ training programs, fostering an active collaboration across the several schools of ULisboa (e.g. Medicine, Pharmacy, Economics and Management, Engineering); the integration of entrepreneurship courses within existing health-related curricula; the development of modular, flexible training programs (e.g. micro-credentials) that allow current professionals to upskill in areas such as digital health or innovation management; the creation of joint training modules or workshops with industry and health organization partners.

## Discussion

In line with the objective of identifying core E&I competencies and aligned training needs (in the form of course topics) for professionals working in the health sector, this study presents the results of a structured multi-stakeholder collaborative approach. While study findings contribute to advance the understanding of how E&I-related education can be systematically integrated into health education curricula, multiple insights can be derived from this study.

### Framing our study within the extant E&I literature

Our findings contribute to advancing the field of health entrepreneurship education by addressing critical gaps identified in previous literature. For instance, while Toner and Tompkins [19] highlighted the importance of fostering innovation within academic medical centres, their work primarily focused on strategies to enhance institutional culture rather than specific competency development. Our study extends this foundation by providing a comprehensive, stakeholder-validated framework that operationalizes entrepreneurial competencies into specific educational content. Building upon the scoping review by Suryavanshi et al. [25], which underscored the fragmented nature of E&I education in health sciences, our research contributes to bridge the link between competency identification and curriculum design. Where previous studies typically addressed either competencies or educational content in isolation, our approach (framed within value-focused thinking) systematically connects these elements, offering a more integrated framework for competency identification and curriculum development. Our work also extends beyond the work of Garbutt et al. [61] by encompassing the broader health sector ecosystem, including healthcare providers, industry, and policy contexts. While their modified Delphi approach validated competencies for biomedical research trainees, our multi-stakeholder approach captures diverse perspectives (healthcare providers, industry, government, and academia) systematically connecting E&I competencies with aligned educational topics.

### Key insights for health education

The identification of 51 E&I competencies across five domains – psychological/self-management, social/interpersonal, organizational, analytical/technical, and health system contextual knowledge – provides a comprehensive foundation for curriculum development. Structuring competencies within this multi-domain framework captures the complex interplay of competencies necessary to address the multifaceted challenges of contemporary healthcare [62]. This study expands previous competency frameworks [e.g. 37, 61, 63] by providing an updated and more comprehensive set of competencies, further tailored to the evolving demands of modern health systems and workplaces. For instance, while the EU EntreComp framework [64] outlines a broad entrepreneurial skillset applicable across all professional sectors, our framework specifically targets the unique health context. Furthermore, our study complements the CanMEDS framework [65], which is specifically designed for physicians and specifies the seven core roles essential for patient-centred care; in contrast, our framework was designed to be generically applicable to all professionals working within the health sector, and emphasizes an orientation towards entrepreneurship and innovation. While a detailed content comparison with established educational models such as CanMEDS is beyond the scope of this study, one can observe that CanMEDS entail physicians’ competencies related with medical expertise, communication, collaboration, leadership, health advocacy, scholarship and professionalism, and does not consider several E&I related competencies such as competencies concerning analytical/technical skills, and health systems and organizational knowledge. A study comparing our results with such frameworks could provide deeper insights into not only which E&I competencies should be integrated, but also how these may be integrated into existing competency-based programs, thereby supporting the adaptation of traditional professional roles to the demands of innovation-driven healthcare systems.

The value-focused thinking approach employed to align 55 course topics with specific E&I competencies provides a novel methodology for curriculum design. Delphi results demonstrated an alignment between E&I competencies and training contents, with 46 out of 55 topics (84%) achieving the 70% relevance agreement threshold. This linkage provides a practical blueprint that can be leveraged by higher education institutions to design and implement competency-based E&I education programs [66], tailored to the specific needs of current and future health professionals. This could involve the creation of standalone courses, the redesign of existing health curricula to incorporate E&I-related courses, or developing entire degree programs specifically focused on ‘E&I in health’. Experiential learning methods - including hackathons [67], or capstone projects [68] – could be employed to promote the development of practical E&I skills, and strategic collaborations between the academia and other health stakeholders should be encouraged to foster knowledge translation, bridging the gap between academic training and real-world healthcare challenges [69]. Also, modular training formats, such as micro-credentials [70] – as suggested in the workshop – could provide flexible upskill pathways for current health professionals.

Variation in agreement rates across stakeholder groups underscores the critical importance of understanding differences in stakeholders’ views, while promoting a consensus. For instance, healthcare providers’ more selective agreement on topic relevance (84% agreement rate), with a lower agreement on business-focused topics — such as ‘Marketing’ (63%) and ‘Business case analysis’ (38%) —, when compared to other groups, may reflect professional cultures that prioritize clinical outcomes and health system effectiveness. Conversely, the health industry group achieved the highest overall agreement (96% agreement rate), especially in business and innovation-related topics. These divergent perspectives highlight that effective E&I curriculum development requires systematic input from diverse stakeholders to ensure relevant educational content that addresses the distinct competency needs of the several professionals working in the health sector.

Furthermore, our study highlights the need interdisciplinary approaches that integrate knowledge from multiple fields, ranging from psychology, business and management, technology, health systems knowledge, among others. This may require establishing formal interdisciplinary collaboration mechanisms between academic departments and creating structured partnerships across diverse disciplines [71] aligning with global calls for curricula that are adaptive, collaborative, and responsive to real-world challenges [18, 72].

The workshop findings complemented the Delphi results by providing contextual insights into implementation challenges and opportunities, particularly within the Portuguese higher education system. Our study produced a comprehensive list of relevant topics that serve as a foundation for future steps, such as fostering collaborative approaches that engage key stakeholders, including representatives from the health industry, health entrepreneurship researchers, and faculty members from various health-related schools at the University of Lisbon (e.g., medicine, nursing, physiotherapy, pharmacy, biomedical engineering, and human kinetics) to discuss the creation of new courses, as well as the adjustment of existing courses. Such collaboration would support the prioritization and selection of E&I curricular topics tailored to each academic program and to workforce needs, involving stakeholders (such as industry partners) with relevance to the specific academic programs.

### Key insights for health policy and organizations

Based on our study findings, several specific policy implications emerge. The high level of agreement among our diverse panel (91% of course topics rated as at least strongly relevant) suggests broad stakeholder support for systematic E&I competency integration in health education, providing a practical tool that policymakers can use to justify investments in E&I curriculum development initiatives.

Furthermore, our study provides insights for advancing human resource management practices within healthcare organizations [73]. Specifically, the 51 E&I competencies identified in the literature review provide a structured and evidence-based framework that can inform recruitment and selection processes, including the creation of competency-based job descriptions, the assessment of potential candidates and the design of tailored interview protocols. Indeed, the five-domain structure allow organizations to prioritize specific competency areas based on their E&I needs and strategic objectives. In addition, the systematic categorization of ‘E&I in health’ competencies offers a foundation for developing workforce performance indicators and competency-based assessment tools [74] to measure employees’ entrepreneurial capabilities and track their development over time. By systematically monitoring E&I competency development over time, health organizations can better support workforce growth in areas critical for fostering innovation and adaptability. Finally, the established link between competencies and course topics makes available a roadmap for designing individualized career development plans. This enables healthcare professionals to identify competency gaps and pursue targeted training opportunities, while organizations can create structured career pathways that formally recognize and reward entrepreneurial competencies and skills as part of professional advancement. Collectively, these insights contribute to a more strategic and competency-driven approach to human resource development in healthcare, supporting the development of a health workforce better equipped to drive innovation and respond to emerging challenges.

### Methodological insights

Regarding the adopted methodological approach, first, the participatory nature of the Delphi process ensured that the competencies and course topics identified (combined with a scoping review) are not only theoretically sound but also practically relevant. The diverse expertise of the 29 participants from academia, healthcare providers, industry, and government enriched the findings, ensuring they resonate with real-world training needs and stakeholder preferences. Second, the application of a VFT framing within the design of Delphi questionnaires along with the integration of the thematic maps into the web-Delphi platform offered a valuable tool for participants to better visualize and understand complex relationships between the different constructs.

### Study limitations

This study has several important limitations that should be acknowledged. First, the scoping review was restricted to peer-reviewed scientific publications and did not consider grey literature, which may have excluded potentially innovative studies or reports from non-academic sources. Moreover, we cannot exclude the possibility of our search strategy may have missed E&I competencies or course topics that are integrated into broader health-related university curricula without being explicitly labelled as “entrepreneurship” or “innovation”.

Second, some considerations on the application of the QCA used in this study are worth discussing. QCA emphasizes the active role of the researcher in analysing and synthesizing data which can introduce subjectivity. Indeed, the process of coding and categories generation may be influenced by the researcher’s own preconceptions potentially overlooking alternative interpretations. While your co-authors’ validation helps reduce any subjective bias, the analysis is still shaped by the collective perspectives, assumptions, and experiences of the research team. Therefore, an alternative categorization of E&I competencies and course topics could also be possible.

Third, we acknowledge the potential for bias introduced by our expert selection criteria in the three-round Delphi process. By intentionally recruiting participants with a specific interest in E&I in health, we likely gathered a panel predisposed to view E&I competencies as particularly relevant, more so than what would be the case with a broader and more representative group of experts.

Fourth, despite there being a careful design of the Delphi questionnaire, namely in its question wording, we cannot exclude the possibility of having occurred response biases, including the primacy effect [75] (i.e. the respondents tend to choose one of the first answer options presented) or the acquiescence bias [76] (participants tendency to lean towards agreement simply to avoid appearing disagreeable or uninformed) or demand characteristics bias [77] (participants picking up clues within the questionnaire adjusting their responses to what they think the research team wants to hear or what they think the research is about). Although less likely given the inherently anonymous nature of the Delphi method and the theme under discussion, social desirability bias [78] (i.e. participants tailoring their answers to fir societal expectations) might also have occurred.

Fifth, an important observation from the Delphi process was the absence of participant comments in Rounds 1 and 2. Several methodological, participant-related, and content-related factors may help explain this outcome. Methodologically, the integration of visual thematic maps directly within the questionnaire may have facilitated comprehension, while the comprehensiveness of Round 0 and the careful wording and structuring of items may have reduced the perceived need for clarification. From the participants’ perspective, time constraints faced by health professionals or cumulative fatigue from completing lengthy closed-ended questionnaires may have discouraged the use of the comment option. Content-related aspects, such as a strong perception of convergence, may also have reduced the perceived value of additional elaboration. Although this pattern could be interpreted as indicative of effective questionnaire design and conceptual clarity, it nonetheless constitutes a limitation. The absence of qualitative feedback restricted access to participants’ underlying reasoning and limited the exploration of nuanced perspectives that the ratings alone could not capture. In particular, it prevented clarification of how participants interpreted items with overlapping scope, and may have masked differences in the rationale underlying similar ratings.

Sixth, the Delphi and workshop phases included only Portuguese participants, which may limit the transferability of findings to other educational, healthcare, and cultural contexts. Cross-country differences in health system organisation can influence the perceived relevance of entrepreneurship and innovation (E&I) competencies; for instance, countries with advanced digital health infrastructures may place greater emphasis on technology-related competencies. Cross-country variations in educational systems and in their governance also play a critical role [79]. Institutions within centralised systems often face stringent regulatory barriers, standardised curricula, and lengthy approval processes, whereas decentralised systems may provide greater flexibility to adapt programmes, pilot innovative approaches, and respond more rapidly to emerging workforce needs. Cultural aspects further shape entrepreneurial attitudes [80] and thus may affect how distinct E&I competencies are prioritised and taught. For example, societies characterised by high uncertainty avoidance or strong hierarchical traditions may require tailored pedagogical strategies to foster risk-taking and leadership skills. Collectively, these considerations indicate that while our framework offers a structured starting point, its application in international contexts requires careful contextualisation and adaptation. Future research should therefore replicate this approach across diverse educational and cultural settings to assess the global relevance of the framework and identify region-specific adaptations.

Finally, regarding the workshop, despite being insightful and informative, other aspects could have been discussed in the workshop, yet due to time issues it was not possible. For instance, it would be interesting to discuss in more detail preferred training formats and the feasibility of different pedagogical strategies for developing health E&I competencies.

### Promising directions for future research

This study establishes several promising directions for future research. Firstly, from a conceptual perspective, subsequent research could enhance the proposed framework by refining the definitions of E&I competencies and establishing proficiency levels to track competency progression. Secondly, multinational Delphi studies could be conducted to validate the global applicability of study findings and identify necessary regional adaptations across different healthcare systems and cultural contexts. Thirdly, comparative studies evaluating different pedagogical strategies and approaches (e.g., hackathons versus capstone projects) could be encouraged to determine optimal strategies for fostering specific E&I competencies among health professionals. Finally, policy research should examine how the identified ‘E&I in health’ competencies can be integrated into existing accreditation frameworks, exploring how health-related professional bodies and higher education accrediting organizations might incorporate E&I competencies into their certification requirements and curriculum standards.

## Conclusion

This study provides a comprehensive, stakeholder-validated list of E&I competencies and aligned course topics for professionals working in the health sector. By systematically bridging ‘E&I in health’ competencies to relevant educational content, study results offer a solid foundation to inform the development of more targeted and practically oriented E&I curricula in health education. From a policy perspective, key recommendations stemming from this research include the urgent need for the integration of ‘E&I in health’ competencies into formal accreditation standards or the allocation of dedicated funding for interdisciplinary ‘E&I in health’ training programs and initiatives. At a managerial level, the comprehensive set of ‘E&I in health’ competencies presented in this study can be used to inform health workforce management practices.

Embedding ‘E&I in health’ competencies and course topics within innovative health workforce planning initiatives can better prepare current and future health professionals with the necessary knowledge, skills, and mindsets to foster innovation and address complex challenges in health systems. As the healthcare landscape continues to evolve rapidly, nurturing these E&I professional competencies will be crucial for developing a workforce capable of leading transformative change and enhancing the overall efficiency of health systems.

## Supplementary Information

Below is the link to the electronic supplementary material.

Supplementary Material 1

Supplementary Material 2

## Data Availability

All data generated and analysed during this study was synthesized and included in this published article (and its supplementary information files). Participants’ individuals responses to Delphi round 0 are available from the corresponding author upon reasonable request.

## Abbreviations

AI: Artificial Intelligence
CVM: Collaborative Value Modelling
E&I: Entrepreneurship & Innovation
EIT: European Institute of Innovation & Technology
EU: European Union
HEI: Higher Education Institutions
PRISMA: Preferred Reporting Items for Systematic Reviews and Meta-Analyses
QCA: Qualitive Content Analysis
ScR: Scoping Review
ULisboa: University of Lisbon
VFT: Value-Focused Thinking
