# Supplementary material for: Preparing tomorrow’s health entrepreneurs: a collaborative multi-stakeholder approach to identifying core competencies and training needs of future professionals
Source: BMC Health Serv Res. 2025 Oct 28;25:1417. doi: 10.1186/s12913-025-13571-2 (PMC12570624; doi:10.1186/s12913-025-13571-2)
Supplement: Supplementary file 1 — Supplementary Material 1 [file 12913_2025_13571_MOESM1_ESM.docx]

# ADDITIONAL FILE 1

Additional file 1 includes:

1. Structured search strategy in different electronic databases;
   1. Characterization of studies included in the scoping review
   2. List of studies included in the scoping review.

## 1.1 Structured search strategy in different electronic databases

Web of Science

TS = ("Health" OR "Medical" OR “Hospital”) AND ("Entrepreneur*" OR "innovation") AND ("course*" OR "train*" OR "curricul*" OR "educat*" OR "competenc*" OR "skill*")

Scopus

TILTLE-ABS-KEY = ("Health" OR "Medical" OR “Hospital”) AND ("Entrepreneur*" OR "innovation") AND ("course*" OR "train*" OR "curricul*" OR "educat*" OR "competenc*" OR "skill*")

EBSCO

Search string: ("Health" OR "Medical" OR “Hospital”) AND ("Entrepreneur*" OR "innovation") AND ("course*" OR "train*" OR "curricul*" OR "educat*" OR "competenc*" OR "skill*")

MEDLINE (via Pubmed)

#1 Search: ("Health"[Title/Abstract] OR "Medical"[Title/Abstract] OR “Hospital”[Title/Abstract]) AND ("Entrepreneur*"[Title/Abstract] OR "innovation"[Title/Abstract])

**#2 Search: ("course*"[Title/Abstract] OR "train*"[Title/Abstract] OR "curricul*"[Title/Abstract] OR "educat*"[Title/Abstract] OR "competenc*"[Title/Abstract] OR “skill*” [Title/Abstract])**

**Search: #1 and #2**

## 1.2 Characterization of studies included in the scoping review

**Table A1 –** Characterization of the studies included in the review.

| **ID** | **First author and year of publication** | **Study title** | **Study objective** | **Study location (country where the study was conducted)** | **Research design** | **Data collection methods** | **Target audience (intended beneficiaries of the competencies and educational topics reported)** | **Competencies reported** | **Topics reported** |
| --- | --- | --- | --- | --- | --- | --- | --- | --- | --- |
| 1 | Sacre et al. (2024) | Self-perceived leadership and entrepreneurship skills: profiling healthcare professionals | Evaluate the self-perception of healthcare professionals regarding leadership and entrepreneurship competencies and their association with demographic characteristics and university attributes using an online cross-sectional survey. | Lebanon | quantitative | cross-sectional survey | healthcare professions in Lebanon: pharmacists, physicians, nurses, dentists, nutritionists, psychologists, physical therapists, medico-social workers, and epidemiologists. | decision-making, strategic thinking, risk taking, confidence building, communicating ideas, motivating team members, tolerance for ambiguity, internal locus of control (from the work of (Rubino, 2005)). | Not reported |
| 2 | da Silva et al. (2023) | Process of building an entrepreneurial career in Nursing | Learn about the experiences of nurse entrepreneurs in building their careers and business trajectories. | Brazil | qualitative | semi-structured interviews | nursing graduates entrepreneurs | creativity, leadership, being persistent, being a self-taught professional, having a vision outside the box, giving things time to happen, be open to new technologies, empowerment (leader rather than a boss), being hard-working, up-to-date and innovative, freedom to discuss experiences. | Not reported |
| 3 | Mohamed et al. (2023) | Born Not Made: The Impact of Six Entrepreneurial Personality Dimensions on Entrepreneurial Intention: Evidence from Healthcare Higher Education Students | Measure entrepreneurial intentions and identify the characteristics and personality dimensions among junior healthcare students and new graduates in Saudi Arabia using a cross-sectional online survey. | Saudi Arabia | quantitative | cross-sectional survey | health entrepreneurs (does not specify) | internal locus of control, innovativeness, autonomy, proactiveness, problem-solving, risk-taking. | Not reported |
| 4 | Dopelt et al. (2023) | I Believe More in the Ability of the Small Person to Make Big Changes: Innovation and Social Entrepreneurship to Promote Public Health in Israel | Explore what motivates public health social entrepreneurs to act, the challenges and barriers they face, achievements, and competencies required for success through interviews with 15 experts in public health entrepreneurship from Israel. | Israel | qualitative | semi-structured interviews | Public health social entrepreneurs in Israel and future social entrepreneurs interested in improving population health. | creativity, perseverance, tolerance, willingness to work hard, risk-taking, flexibility, dealing with situations of uncertainty | Not reported |
| 5 | Xu et al. (2022) | A modified Delphi study on establishing a curriculum content structure for the leadership and management competency cultivation for future nurse managers in China | Establish a curriculum content structure for the leadership and management competency cultivation for future nurse managers in Chinese healthcare setting. | China | mixed methods | Initial curriculum content was based on a qualitative study and team meetings. Two-round Delphi survey with experts to reach consensus | nurse managers | Not reported | 1. Fundamentals of nursing leadership and management 2. Intra-and interprofessional teamwork 3. Healthcare policy and law 4. Human resources and position management 5. Quality of care and patient safety 6. Evidence-based practice 7. Healthcare cost management 8. Informatics 9. Public health emergency response |
| 6 | Thompson et al. (2022) | Training the next generation of translational scientists: The Case Western Reserve University Translational Fellows Program | Present a training program in I&E for biomedical scientists and trainees. | USA | descriptive research (Program description) | not applied | graduate students and postdoctoral fellows, specifically senior doctoral students and postdocs in biomedical or health-oriented fields | Not reported | Intellectual Property technology transfer, identifying your market potential, identifying sources of funding, pitching your technology. |
| 7 | Scahill et al. (2022) | The pharmacist as entrepreneur: Whether, how, and when to educate? | Explore the views of community pharmacists and key sector stakeholders about personal experience of entrepreneurship and business-related training; investigate their views on whether entrepreneurship training is needed and how, when, and by whom it should be delivered. | New Zealand | exploratory, qualitative | semi-structured interviews | Community pharmacists and key sector stakeholders in the pharmacy sector | problem solving, critical thinking, leadership skills, adaptability, self-assessment, dealing with failure, and communication. | Not reported |
| 8 | Grailer et al. (2022) | A Novel Innovation and Entrepreneurship (I&E) Training Program for Biomedical Research Trainees | Present a program - Entrepreneurship for Biomedicine (E4B) program – that was created to develop biomedical researchers’ I&E skills. The program is housed at Washington University School of Medicine in St. Louis and is open to pre- and postdoctoral biomedical research trainees and faculty nationwide. | USA | descriptive research (Program description) | nor reported | Pre- and postdoctoral biomedical research trainees | Not reported | Module 1 of the course: Introduction to the World of Biomedical I&E, Survive and Thrive as an Innovator (psychological well-being, including self-esteem, gratitude, the mind– body connection, physical activity, emotional intelligence, and resilience), identifying Opportunities or Innovation, Building Effective Teams, Diversity, Equity, and Inclusion in I&E, Validating Your Innovation, Fishing for customers, ethics, selling your innovation. Module 2 of the course: funding your innovation, intellectual property, sustaining and scaling your innovation, painting a defensible picture of the future, defining your product, and managing development, leadership skills, communication skills. |
| 9 | Cuddihy et al. (2021) | A program to drive innovation and entrepreneurship in academic cardiovascular center incorporating clinical team and patient codesign | Present an innovation academy for early-stage innovation and entrepreneurship education, involving faculty, trainees, and staff, patients and family members. | USA | case study/program evaluation | Pre-and post-course surveys that included self-assessments | Clinical and Research Faculty, Medical Trainees and Residents, Healthcare Staff, Patients and Family Members, Healthcare Innovators and Entrepreneurs, Hospital and Health System Administrators, Technology Transfer and Commercialization Professionals | Not reported | Introduction to intellectual property, customer discovery, stakeholders & markets, regulatory process, stakeholders, features and benefits, customer discovery reports, Communicating Innovation, Introduction to Pitch Template, Planning & Development Milestone, Equity & Investments, pitch presentations, pitch showcase. |
| 10 | Chan et al. (2021) | For Students, by Students: a Peer-Led Entrepreneurship Course for Medical Students | Present the structure of an elective module on medical innovation and entrepreneurship designed by final year UK medical students. | UK | descriptive research (Program description and evaluation) | questionnaires to assess student feedback on the course | medical undergraduate students | Not reported | Course structure: Introduction to Innovation and Leadership, Quality improvement 1, Quality Improvement 2, Medical Technology, medical education and technology, entrepreneurship and leadership, leadership, assessment and presentation. |
| 11 | Afeli et al. (2021) | Curriculum content for innovation and entrepreneurship education in US pharmacy programs | Assess similarities and differences in curricular content between entrepreneurship and innovation training programs in the USA and to present a set of must-haves for innovation and entrepreneurship curricular content for core curricula in pharmacy. | USA | qualitative | Document analysis and direct inquiry (contacting program administrators) | Pharmacy students and pharmacists | Not reported | Key topics such as financial management, marketing, leadership, organizational behaviour, and human resource management; entrepreneurship, accounting, innovation, management |
| 12 | Aceituno-Aceituno et al. (2021) | Scientific Mobility, Training and Entrepreneurial Skills in Health Sciences: The Spanish Case | Provide and discuss data on the training received in entrepreneurship by Spanish scientific mobility workers in Health Science, their acquisition of entrepreneurial skills, and the encouragement of their entrepreneurial and intrapreneurial intentions. | Spain | quantitative | cross-sectional survey | scientists in health sciences | positive mental attitude, ability to overcome failure, code of ethics, meting management, stress management, social relations, time management, conversation skills, proactive attitude, leadership, employee motivation, negotiation, organization and delegation, planning, personnel selection, perseverance, foresight and project for the future. | Not reported |
| 13 | Suryavanshi et al. (2020) | Entrepreneurship and Innovation in Health Sciences Education: a Scoping Review | Scoping review aimed to explore the connection between health education and entrepreneurship and to identify gaps in the current literature, educational models, and best practices regarding teaching medical professionals about entrepreneurship and innovation. | not applied | scoping review | scoping review | health sciences students, health professionals, entrepreneurs | Not reported | Major themes identified: Securing funding and capital and commercialization of ideas, idea generation, the principles of design thinking and how to generate and maintain an effective team, intellectual property laws, understanding regulation. |
| 14 | Panther et al. (2019) | Addressing Unmet Patient Care Needs Through Curricular Development of Student Pharmacist Leadership and Entrepreneurial Skills | Present the implementation and evaluation results of a curriculum designed to increase student pharmacists’ skills and confidence in addressing unmet patient care needs and the Institute for Healthcare Improvement’s (IHI) Triple Aim goals in the community pharmacy setting. | USA, multi-site | quantitative | pre-course, post-course, and retrospective assessments to measure changes in students' confidence and skills. | Pharmacy students and educators | Not reported | Leadership, teamwork, Kotter’s 8 steps for leading change, project pitch delivered to classmates, faculty, and pharmacy partners. |
| 15 | Mattingly et al. (2019) | A Systematic Review of Entrepreneurship in Pharmacy Practice and Education. American Journal of Pharmaceutical Education | Review literature pertaining to entrepreneurship in pharmacy practice, education, and the knowledge, skills, and attitudes identified for pharmacist entrepreneurs. | not applied | systematic review | systematic review | Pharmacy students, educators, and practicing pharmacists | risk-taking, creativity/innovation, self-starter, management, proactivity, communication, strategic planning, positivity, decision-making, teamwork, versatility, marketing, critical thinking, competitiveness, proposal development, numeracy, technology, self-reflection, persistence, social responsibility, cultural competence. | Not reported |
| 16 | Garbutt et al. (2019) | Validating curricular competencies in innovation and entrepreneurship for biomedical research trainees: A modified Delphi approach | Identify competencies for I&E training for biomedical researchers and to identify program content though a modified Delphi process with six panels. One panel validated the appropriateness of the competencies reported in the EU EntreComp framework for the field of health. The other 5 panels identified 120 topics to be included in a training curriculum. | USA | mixed methods | literature review + Delphi | biomedical research trainees, biomedical scientists | Management: planning and management, financial and economic literacy, mobilizing resources; Vision and imagination: spotting opportunities, vision, valuing ideas, creativity; Social skills: self-awareness and self-efficacy, mobilizing others, working with others; Psychological skills: learning through experience, taking the initiative, motivation and perseverance; Ethical and decision-making skills: coping with uncertainty, ambiguity and risk, ethical and sustainable thinking. | Not reported |
| 17 | McGloughlin et al. (2018) | Innovation for the future of Irish MedTech industry: retrospective qualitative review of impact of BioInnovate Ireland's clinical fellows | Identify and describe the core competencies and skills considered essential for success of pharmacists though a facilitated workshop; examine the impact of BioInnovate Ireland has had on the clinicians involved and validate the collaborative process. | Ireland | descriptive research (Program description and evaluation) | cross-sectional survey | health professionals mainly clinicians | critical thinking and problem solving; collaboration across networks and leading by influence; agility and adaptability; initiative and entrepreneurialism; effective oral and written communication; accessing and analysing information; curiosity and imagination; and self-awareness. | Not reported |
| 18 | Servoss et al. (2018) | The Surgery Innovation and Entrepreneurship Development Program (SIEDP): An Experiential Learning Program for Surgery Faculty to Ideate and Implement Innovations in Health care | Present an experiential learning program specifically designed for clinical and research faculty in a major academic surgery department. The program is called SIEDP (e Surgery Innovation and Entrepreneurship Development Program). | USA | descriptive research (Program description and evaluation) | post-course online survey to assess student feedback on the course | Clinical and research faculty in university surgery departments | Not reported | The curriculum includes a range of topics, yet those related to entrepreneurship and innovation include:  Trends in medical innovation, developing innovation projects (facilitated ideation session), how to conduct a business pitch, project selection and team formation, value proposition design and analysis, the innovation landscape: developing stakeholder maps, Case Study: The Importance of a Value Proposition, Customer Development (Discovery): The Best Approach for Quality Interviews, Risk as an Effective Planning Strategy, Building Your Presentation: The Executive Summary Template, Intellectual Property and Paths to Commercialization, Product/Project Development Planning, Conducting Customer Development (Discovery) Interviews, Medical and Engineering Collaborations, Health care Economics: Supporting Data for your Innovation Project, Industry Engagement, Change at the Food and Drug Administration, pitch presentation coaching, final pitch event and external expert feedback. |
| 19 | Prado et al. (2018) | Management training in global health education: a Health Innovation Fellowship training program to bring healthcare to low-income communities in Central America | Assess the impact training program – Health Innovation Fellowship - for practicing health professionals offered jointly by the Central American Healthcare Initiative and INCAE Business School, Costa Rica. | Costa Rica | descriptive research (Program description and evaluation) | surveys and document analysis | Practicing health professionals in Central America, particularly those involved in addressing health disparities and working in rural communities. | Not reported | module I: leadership for healthcare innovation, Determinants of health, Innovation and healthcare, team building, Global Health; module II: networking and social networks, project management, finance for project management, organizational change; module III: networking: public-private alliances, effective communication, political analysis, negotiation, healthcare in politically unstable countries, human resource management, health economics, Intersectoral Alliances/Replication of Social Projects. |
| 20 | Amini et al. (2018) | Identifying social entrepreneurship competencies of managers in social entrepreneurship organizations in healthcare sector | Identify the social entrepreneurship competencies of managers in social entrepreneurship organizations in the healthcare sector through a series of interviews. Develop a competency model for social entrepreneurship training for health managers. | Iran | qualitative | semi-structured interviews | managers of social entrepreneurship organizations in the healthcare sector | Communication: networking, effective communication, negotiation. Individual competencies: perseverance and pursuit, self-development, changeability, collectivism, internal locus of control, self-knowledge, strategic thinking, problem solving, creativity.  Managerial competencies: planning, organizing, monitoring & controlling, human resource management, leadership, teamwork, empowerment (develop others), strategic planning, project management, knowledge management, financial management. Social competencies: trust commitment to help the deprived, empathy and sympathy, social concerns, social culture, developing social participation, ability to recognize social problems.  Health professional entrepreneurial competencies: being influenced by role model in the field of health, entrepreneurial intention in the field of health, benefit from others’ experiences, previous experience, Specific and technical knowledge, and education in the field of health, ability to analyse the health business environment, ability to identify opportunities, ability for financing, ability to recognize target groups in the field of health, ability to recognize the requirements of target groups in the field of health. | Not reported |
| 21 | Niccum et al. (2017) | Innovation and entrepreneurship programs in US medical education: a landscape review and thematic analysis | Characterize innovation and entrepreneurship education in US medical schools to provide insight into the features, skills listed and learning objectives, using thematic analysis from public accessible resources. | USA | mixed methods | systematic searches of medical school websites, document analysis, interviews with program directors, survey to collect program characteristics, thematic analysis the identify common curricular themes | educators and administrators of medical schools who intend to launch a innovation and entrepreneurship programs at their higher education institutions | Example of skills related to 'Innovation': idea creation, navigating complex environments, interdisciplinary problem solving. Example of skills related to 'Entrepreneurship': business planning, executive leadership, interdisciplinary teamwork, and financial management. Example skills listed as related to leadership included leadership styles, change management, conflict resolution, inter-professional team dynamics, [and] use of organizational management tools’. Example skills related to 'technology' include product development, needs identification, and prototype design. Example skills related to 'healthcare systems' include systems-based practice, advocacy. Example skills related to 'business of medicine': general under-standing of financial reimbursement, the application of new reimbursement models such as value-based care, and the creation of financial models to justify investment opportunities. | Not reported |
| 22 | Cohen (2017) | Enhancing surgical innovation through a specialized medical school pathway of excellence in innovation and entrepreneurship: Lessons learned and opportunities for the future. Surgery. | Describe the creation and implementation of a specific program at the University of Michigan Medical School, known as the Pathway of Excellence in Innovation and Entrepreneurship. | USA | descriptive research (Program description) | not reported | medical students | Not reported | Topics covered in the curriculum included the following: concept brainstorming; clinical decision trees and ecosystem mapping; project formation; development of strong value propositions; reimbursement strategies; market sizing; customer discovery; introduction to health economics, hospital economics, and purchasing decisions; regulatory pathways and the Food and Drug Administration; overview of intellectual property, including prior art searches, alternative protection, and freedom to operate; development of a strong intellectual property strategy; creation of a viable regulatory strategy; clinical trial design, medical device, and information technology development; early prototyping and preclinical proof of concept; preapproval regulatory requirements; how to run a company; funding strategies and opportunities for startups; equity and stock options 101; how to give a compelling pitch; and human factors of error analysis. |
| 23 | White et al. (2016) | Nurse leaders and the innovation competence gap | Assess critical competencies for innovation success among nurse leaders in academia and practice through a web-based self-assessment survey; identify perceived gaps in those competencies; determine teaching methods that would be helpful in developing competencies related to innovation. | USA | quantitative | cross-sectional survey to capture nurse leaders' perceptions of important innovation competencies and how they assess their level of competence in the particular innovation domain. | nursing professionals | the ability to recognize an opportunity, the ability to assess the feasibility of an opportunity, risk management/mitigation, ability to convey a compelling vision, tenacity and perseverance, creativity problem-solving/imaginativeness, the ability to leverage resources/bootstrapping, guerilla skills/use of unconventional approaches, ability to focus yet adapt, resilience, design thinking, self-efficacy/confidence, building and using networks, change management, understanding of health care systems, cross disciplinary knowledge, information management, understanding of behavioural economics, interdisciplinary teamwork and collaboration. | Not reported |
| 24 | Ramia et al. (2016) | Mapping and assessment of personal and professional development skills in a pharmacy curriculum | Assess whether the personal and professional development subdomains are integrated in a pharmacy curriculum at the Lebanese American University School of Pharmacy. | Lebanon | mixed methods | surveys and document analysis | pharmacy students, educators and professionals | management, self-awareness, creativity/innovation, self-starter, versatility. | Not reported |
| 25 | Laverty et al. (2015) | Developing Entrepreneurial Skills in Pharmacy Students | Present the implementation and evaluation of a workshop that teaches undergraduate pharmacy students about entrepreneurship. | UK | quantitative | surveys | pharmacy students, educators and professionals | numeracy, proposal development, critical thinking, communication, management. | Not reported |
| 26 | Salminen et al. (2014) | Entrepreneurship Education in Health Care Education | This study addresses the issue by describing entrepreneurship education in six different Finnish polytechnics. Based on a survey conducted among teachers in the health care field, the study focuses on the scope and nature of entrepreneurship education as well as on the methods that teachers use when addressing the issue of entrepreneurship. | Finland | mixed methods | surveys | students, educators and administrators of health science schools | Not reported | Content of entrepreneurship education: establishment of a company (business idea and plan), entrepreneurship in general, marketing, how to become an entrepreneur, production, quality, leadership, legislation, customer orientation, financial management, networking, ethics of entrepreneurship |
| 27 | Boore et al. (2011) | Education for entrepreneurship in nursing | Present and discuss the integration of a strategy for development of entrepreneurship education within a region of the UK within a nursing program. | UK | case study/program description | not mentioned | nursing students | Not reported | The program is structured around six key themes, with specific emphasis on Leadership and Management, and Research and Evidence Informed Practice. Year 1 covers foundational topics like creativity, innovation, needs assessment, planning, delivering care, and e-learning. Year 2 includes Decision-Making in Multidisciplinary Practice, team characteristics, and Public Health needs assessment. Year 3 focuses on Management of Change, introducing innovation into practice. |
| 28 | Guo (2009) | Core competencies of the entrepreneurial leader in health care organizations | Discuss core competencies in entrepreneurship and leadership for health leaders from the literature, and proposes a competency model. | not applied | literature review | literature review | clinicians in leadership positions in healthcare organizations, managers working in the health sector | Interpersonal competencies: communication, self-development, motivation. Health care system and environment competencies: knowledge of the health system and regulation, alignment with needs and values of the organization, strategic planning. Internal organization competencies: ability to apply complex concepts, develop creative solutions; ability to understand and use statistical, financial, and outcome-based methods to set goals and measure organizational performance. Organizational awareness (ability to understand and learn the formal and informal decision-making structures and power relationships in the organization and with stakeholders). | Not reported |
| 29 | Rubino et al. (2005) | Developing entrepreneurial competencies in the healthcare management undergraduate classroom | Identify clusters of entrepreneurial competencies and training methodologies that can be applied in the classroom to enhance entrepreneurial skills in undergraduate health administration students. | not applied | qualitative | literature review and survey to measure students' self-perception of their entrepreneurial competencies | health administrators and managers | decision-making, strategic thinking, risk taking, confidence building, communicating ideas, motivating team members, tolerance for ambiguity, internal locus of control. | Not reported |

## 1.3 List of studies included in the review

Studies included in the review were categorized in two different groups: group A comprises studies that report on competencies related to entrepreneuship and innovation (E&I) in health, while group B include studies that report course topics/subjects associated to E&I in health that have been addressed and included in higher education health sciences curricula.

**Group A studies (N=16)**

Sacre H, Iskandar K, Haddad C, Shahine M, Hajj A, Zeenny RM, et al. Self-perceived leadership and entrepreneurship skills: profiling healthcare professionals. Journal of Pharmaceutical Health Services Research. 2024;15(1):1-11.

Mohamed ME, Elshaer IA, Azazz AMS, Younis NS. Born Not Made: The Impact of Six Entrepreneurial Personality Dimensions on Entrepreneurial Intention: Evidence from Healthcare Higher Education Students. Sustainability. 2023;15(3):2266.

Dopelt K, Mordehay N, Goren S, Cohen A, Barach P. "I Believe More in the Ability of the Small Person to Make Big Changes": Innovation and Social Entrepreneurship to Promote Public Health in Israel. Eur J Invest Health Psychol Educ. 2023;13(9):1787-800.

da Silva VLD, Spigolon DN, Peruzzo HE, Costa MAR, Souza VS, Christinelli HCB, et al. Process of building an entrepreneurial career in Nursing. Rev Esc Enferm USP. 2023;57:e20230086.

Scahill SL, D'Souza NJ. The pharmacist as entrepreneur: Whether, how, and when to educate? Curr Pharm Teach Learn. 2022;14(1):5-12.

Aceituno-Aceituno P, Danvila-del-Valle J, García AG, Bousoño-Calzón C. Scientific Mobility, Training and Entrepreneurial Skills in Health Sciences: The Spanish Case. International Journal of Environmental Research and Public Health. 2021;18(4).

Garbutt J, Antes A, Mozersky J, Pearson J, Grailer J, Toker E, et al. Validating curricular competencies in innovation and entrepreneurship for biomedical research trainees: A modified Delphi approach. J Clin Transl Sci. 2019;3(4):165-83.

Mattingly TJ, Mullins CD, Melendez DR, Boyden K, Eddington ND. A Systematic Review of Entrepreneurship in Pharmacy Practice and Education. American Journal of Pharmaceutical Education. 2019;83(3):7233.

Amini Z, Arasti Z, Bagheri A. Identifying social entrepreneurship competencies of managers in social entrepreneurship organizations in healthcare sector. Journal of Global Entrepreneurship Research. 2018;8(1).

McGloughlin EK, Anglim P, Keogh I, Sharif F. Innovation for the future of Irish MedTech industry: retrospective qualitative review of impact of BioInnovate Ireland's clinical fellows. BMJ Innov. 2018;4(1):32-8.

Niccum BA, Sarker A, Wolf SJ, Trowbridge MJ. Innovation and entrepreneurship programs in US medical education: a landscape review and thematic analysis. Med Educ Online. 2017;22(1):1360722.

White KR, Pillay R, Huang X. Nurse leaders and the innovation competence gap. Nurs Outlook. 2016;64(3):255-61.

Ramia E, Salameh P, Btaiche IF, Saad AH. Mapping and assessment of personal and professional development skills in a pharmacy curriculum. BMC Med Educ. 2016;16(1):19.

Laverty G, Hanna LA, Haughey S, Hughes C. Developing Entrepreneurial Skills in Pharmacy Students. Am J Pharm Educ. 2015;79(7):106.

Guo KL. Core competencies of the entrepreneurial leader in health care organizations. Health Care Manag (Frederick). 2009;28(1):19-29.

Rubino L, Freshman B. Developing entrepreneurial competencies in the healthcare management undergraduate classroom. The Journal of health administration education. 2005;22(4):399-416.

**Group B studies (N=13)**

Xu X, Zhang Y, Zhou P, Lin Y, Pan W. A modified Delphi study on establishing a curriculum content structure for the leadership and management competency cultivation for future nurse managers in China. Heliyon. 2022;8(12):e12183.

Grailer JG, Alhallak K, Antes AL, Kinch MS, Woods L, Toker E, et al. A Novel Innovation and Entrepreneurship (I&E) Training Program for Biomedical Research Trainees. Acad Med. 2022;97(9):1335-40.

Thompson CL, Misko TA, Chance MR. Training the next generation of translational scientists: The Case Western Reserve University Translational Fellows Program. J Clin Transl Sci. 2022;6(1):e47.

Afeli SA, Adunlin G. Curriculum content for innovation and entrepreneurship education in US pharmacy programs. Industry and Higher Education. 2021;36(1):13-8.

Cuddihy MJ, Servoss JM, Olson DC, Martin BJ, Vemuri C, Eagle KA, et al. A program to drive innovation and entrepreneurship in academic cardiovascular center incorporating clinical team and patient codesign. Journal of Clinical and Translational Science. 2021;5(1).

Chan SCC, Choa G, Eboreime O, Rashid MA. For Students, by Students: a Peer-Led Entrepreneurship Course for Medical Students. Med Sci Educ. 2021;31(6):1735-7.

Suryavanshi T, Lambert S, Lal S, Chin A, Chan TM. Entrepreneurship and Innovation in Health Sciences Education: a Scoping Review. Med Sci Educ. 2020;30(4):1797-809.

Panther SG, Allen RA, Brantner K, Jefferson CG, Murphy NL, Robinson JD. Addressing Unmet Patient Care Needs Through Curricular Development of Student Pharmacist Leadership and Entrepreneurial Skills. American Journal of Pharmaceutical Education. 2019;83(5):840-50.

Prado AM, Pearson AA, Bertelsen NS. Management training in global health education: a Health Innovation Fellowship training program to bring healthcare to low-income communities in Central America. Glob Health Action. 2018;11(1):1408359.

Servoss J, Chang C, Olson D, Ward KR, Mulholland MW, Cohen MS. The Surgery Innovation and Entrepreneurship Development Program (SIEDP): An Experiential Learning Program for Surgery Faculty to Ideate and Implement Innovations in Health care. J Surg Educ. 2018;75(4):935-41.

Cohen MS. Enhancing surgical innovation through a specialized medical school pathway of excellence in innovation and entrepreneurship: Lessons learned and opportunities for the future. Surgery. 2017;162(5):989-93.

Salminen L, Lindberg E, Gustafsson ML, Heinonen J, Leino-Kilpi H. Entrepreneurship Education in Health Care Education. Education Research International. 2014; 2014:1-8.

Boore J, Porter S. Education for entrepreneurship in nursing. Nurse Educ Today. 2011;31(2):184-91.
