# Supplementary material for: Preparing tomorrow’s health entrepreneurs: a collaborative multi-stakeholder approach to identifying core competencies and training needs of future professionals
Source: BMC Health Serv Res. 2025 Oct 28;25:1417. doi: 10.1186/s12913-025-13571-2 (PMC12570624; doi:10.1186/s12913-025-13571-2)
Supplement: Supplementary file 2 — Supplementary Material 2 [file 12913_2025_13571_MOESM2_ESM.docx]

# ADDITIONAL FILE 2 – INFORMATION CONCERNING THE DELPHI PROCESS

Additional file 2 includes:

2.1 Delphi questionnaire screens (examples for Round 2 and for the feedback survey);

2.2. Refined thematic map with final list ‘E&I in health’ competencies (derived from the synthesis of participants’ answers to Round 0;

2.3. Refined ‘E&I in health’ competencies thematic map (derived from the analysis and synthesis of participants’ answers to Round 0);

2.4. Web-Delphi Round 1 distribution of results;

2.5. Web-Delphi Round 2 distribution and analysis of results by stakeholder group;

2.6. Opinion changes between Delphi rounds 1 and 2;

2.7. Results of the feedback survey (following completion of Round 2 of the web-Delphi process).

#
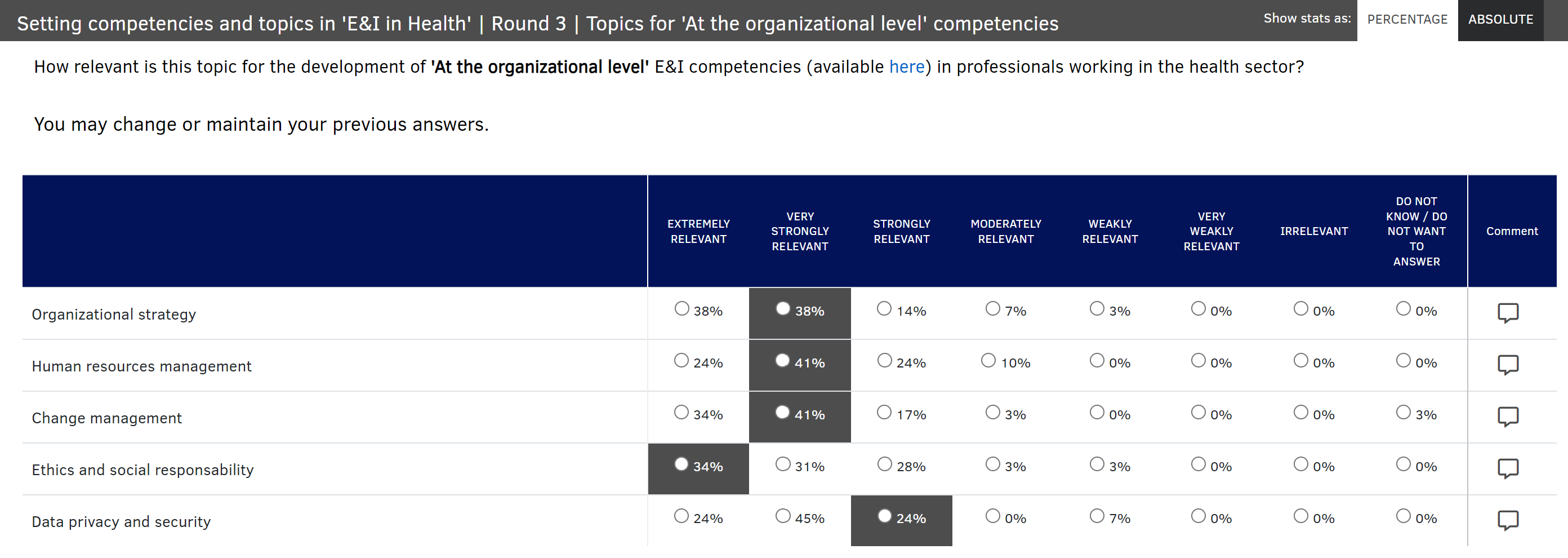
2.1 Delphi questionnaire screens (examples for Round 2 and for the feedback survey)

**Fig. S1** - Example of Round 2 questionnaire screen (screenshot from the Welphi platform). In this screen, participants’ could see a statistical summary of all participants’ answers concerning the relevance of each topic for contributing to the development of ‘At the organizational level’ E&I competencies’. In light of this information, participants change or keep their previous answer (highlighted in grey).


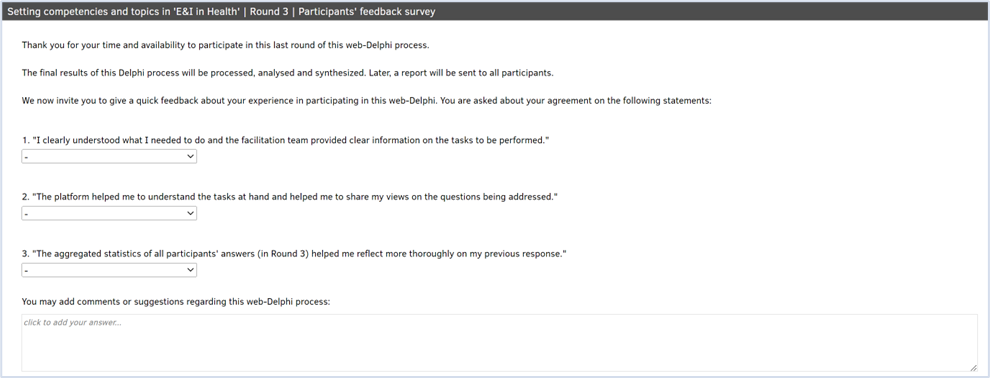


**Fig. S2** – Participants feedback survey to be answered after completion of the Delphi process (screenshot from the Welphi platform).

# 2.2. Refined ‘E&I in health’ competencies thematic map (derived from the analysis and synthesis of participants’ answers to Round 0);


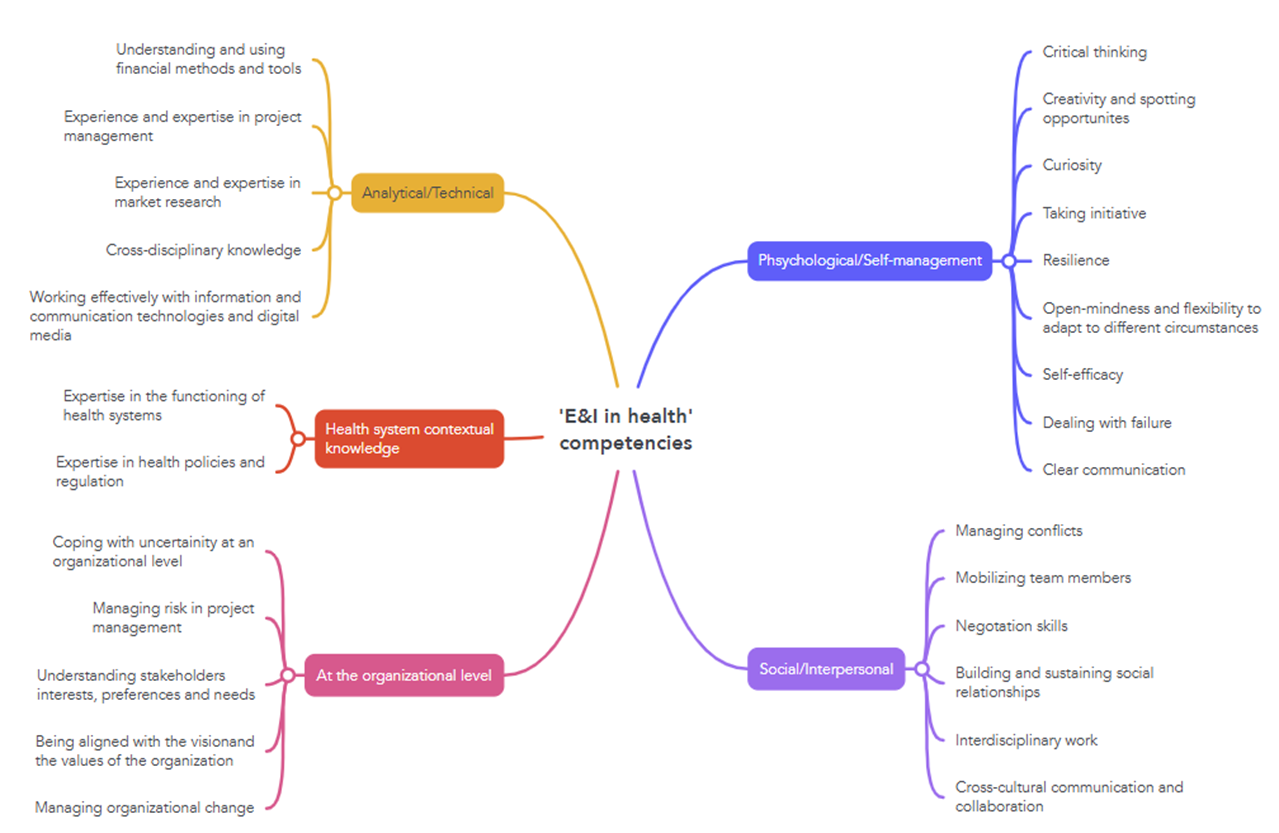


**Fig. S3** Refined ‘E&I in health’ competencies thematic map, updated following the analysis and synthesis of participants’ answers to Delphi round 0. This thematic map was embedded in the web-Delphi platform and served as input information for Delphi round 1.

# 2.3. Refined ‘E&I in health’ course topics thematic map (derived from the analysis and synthesis of participants’ answers to Round 0);


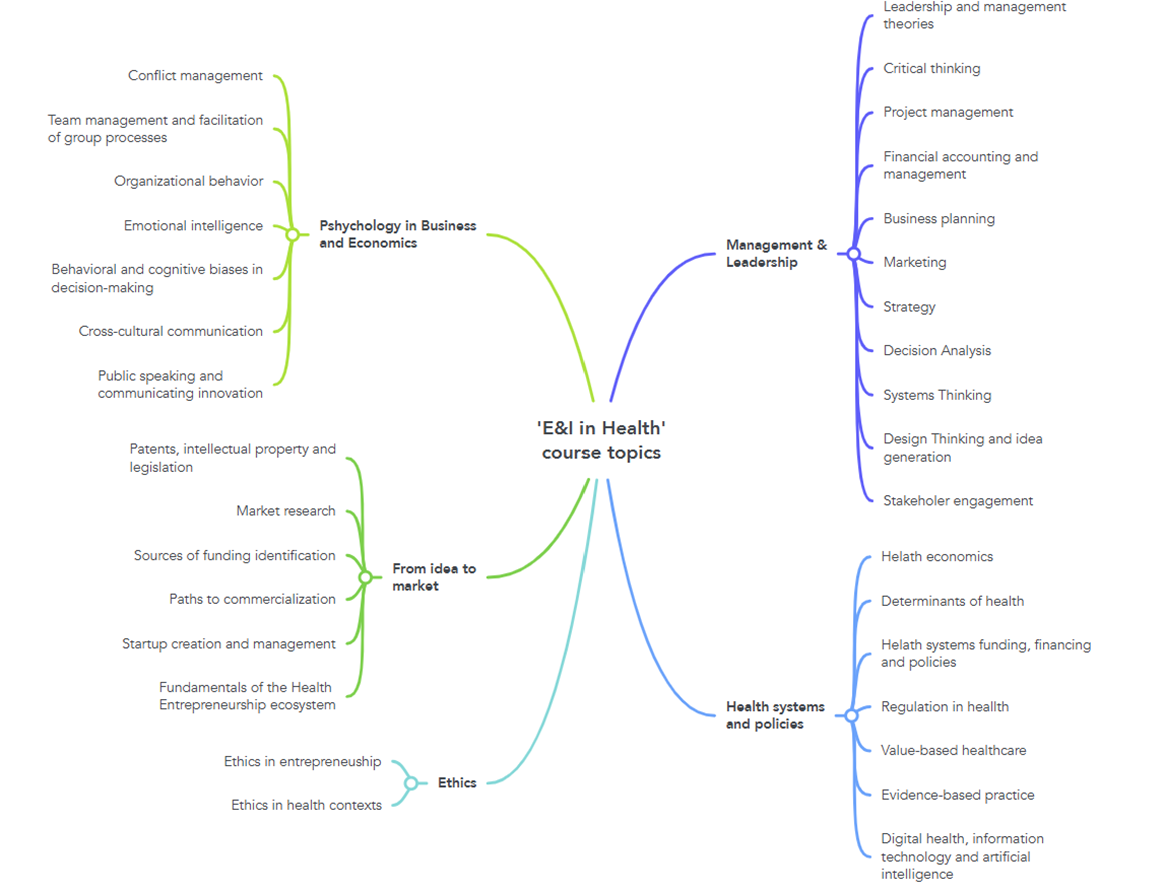


**Fig. S4** Refined ‘E&I in health’ course topics thematic map, updated following the analysis and synthesis of participants’ answers to Delphi round 0. This updated list of course topics was presented in Delphi subsequent rounds (rounds 1 and 2).

# 2.4 Web-Delphi Round 1 distribution of results

**Table S2** Web-Delphi Round 1 distribution of panel results (N=29 participants).

| **‘PSYCHOLOGICAL/SELF-MANAGEMENT’ E&I COMPETENCIES**  **COURSE TOPICS** | **EXTREMELY**  **RELEVANT** | **VERY STRONGLY**  **RELEVANT** | **STRONGLY**  **RELEVANT** | **MODERATELY**  **RELEVANT** | **WEAKLY**  **RELEVANT** | **VERY WEAKLY RELEVANT** | **IRRELEVANT** | **DO NOT KNOW/DO NOT WANT TO ANSWER** |
| --- | --- | --- | --- | --- | --- | --- | --- | --- |
| Behavioural and cognitive biases in decision-making | 28% | 34% | 24% | 10% | 3% | 0% | 0% | 0% |
| Know Thyself (Psychology of Personality) | 17% | 14% | 55% | 10% | 0% | 3% | 0% | 0% |
| Emotional Intelligence | 31% | 41% | 24% | 3% | 0% | 0% | 0% | 0% |
| Mental health and well-being | 52% | 24% | 17% | 3% | 0% | 3% | 0% | 0% |
| Professional resilience | 31% | 34% | 21% | 10% | 0% | 3% | 0% | 0% |
| Critical Thinking | 62% | 21% | 14% | 3% | 0% | 0% | 0% | 0% |
| Risk taking and dealing with failure | 38% | 38% | 21% | 3% | 0% | 0% | 0% | 0% |
| Time and stress management | 31% | 28% | 34% | 3% | 3% | 0% | 0% | 0% |
| **‘SOCIAL/INTERPERSONAL’ E&I COMPETENCIES**  **COURSE TOPICS** | **EXTREMELY**  **RELEVANT** | **VERY STRONGLY**  **RELEVANT** | **STRONGLY**  **RELEVANT** | **MODERATELY**  **RELEVANT** | **WEAKLY**  **RELEVANT** | **VERY WEAKLY RELEVANT** | **IRRELEVANT** | **DO NOT KNOW/DO NOT WANT TO ANSWER** |
| Leadership and management theories | 31% | 24% | 24% | 14% | 3% | 3% | 0% | 0% |
| Public speaking and communication | 21% | 45% | 28% | 7% | 0% | 0% | 0% | 0% |
| Media training | 3% | 21% | 34% | 24% | 14% | 3% | 0% | 0% |
| Stakeholder engagement | 45% | 24% | 21% | 10% | 0% | 0% | 0% | 0% |
| Organizational behaviour and social psychology | 17% | 34% | 31% | 7% | 7% | 3% | 0% | 0% |
| Team management and facilitation of group processes | 48% | 24% | 24% | 3% | 0% | 0% | 0% | 0% |
| Negotiation | 31% | 31% | 38% | 0% | 0% | 0% | 0% | 0% |
| Conflict management | 41% | 31% | 24% | 3% | 0% | 0% | 0% | 0% |
| Cross-cultural communication | 17% | 31% | 38% | 14% | 0% | 0% | 0% | 0% |
| **‘AT THE ORHANIZATIONAL LEVEL’ E&I COMPETENCIES**  **COURSE TOPICS** | **EXTREMELY**  **RELEVANT** | **VERY STRONGLY**  **RELEVANT** | **STRONGLY**  **RELEVANT** | **MODERATELY**  **RELEVANT** | **WEAKLY**  **RELEVANT** | **VERY WEAKLY RELEVANT** | **IRRELEVANT** | **DO NOT KNOW/DO NOT WANT TO ANSWER** |
| Organizational strategy | 38% | 38% | 14% | 7% | 3% | 0% | 0% | 0% |
| Human resources management | 24% | 41% | 24% | 10% | 0% | 0% | 0% | 0% |
| Change management | 34% | 41% | 17% | 3% | 0% | 0% | 0% | 3% |
| Ethics and social responsibility | 34% | 31% | 28% | 3% | 3% | 0% | 0% | 0% |
| Data privacy and security | 24% | 45% | 24% | 0% | 7% | 0% | 0% | 0% |
| **‘ANALYTICAL/TECHNICAL’ E&I COMPETENCIES**  **COURSE TOPICS** | **EXTREMELY**  **RELEVANT** | **VERY STRONGLY**  **RELEVANT** | **STRONGLY**  **RELEVANT** | **MODERATELY**  **RELEVANT** | **WEAKLY**  **RELEVANT** | **VERY WEAKLY RELEVANT** | **IRRELEVANT** | **DO NOT KNOW/DO NOT WANT TO ANSWER** |
| Project management | 34% | 34% | 24% | 7% | 0% | 0% | 0% | 0% |
| Financial accounting and management | 10% | 14% | 45% | 28% | 3% | 0% | 0% | 0% |
| Business planning | 14% | 28% | 55% | 3% | 0% | 0% | 0% | 0% |
| Marketing | 21% | 7% | 38% | 28% | 7% | 0% | 0% | 0% |
| Decision analysis | 21% | 31% | 38% | 7% | 3% | 0% | 0% | 0% |
| Systems thinking | 28% | 24% | 34% | 7% | 3% | 0% | 0% | 3% |
| Risk management | 24% | 34% | 31% | 3% | 3% | 3% | 0% | 0% |
| Quality management | 3% | 41% | 24% | 28% | 0% | 3% | 0% | 0% |
| Lean management | 7% | 34% | 28% | 21% | 7% | 0% | 0% | 3% |
| Design thinking and idea generation | 28% | 28% | 31% | 7% | 7% | 0% | 0% | 0% |
| Data analytics, big data and artificial intelligence | 21% | 24% | 48% | 7% | 0% | 0% | 0% | 0% |
| **‘ANALYTICAL/TECHNICAL’ E&I COMPETENCIES**  **COURSE TOPICS** | **EXTREMELY**  **RELEVANT** | **VERY STRONGLY**  **RELEVANT** | **STRONGLY**  **RELEVANT** | **MODERATELY**  **RELEVANT** | **WEAKLY**  **RELEVANT** | **VERY WEAKLY RELEVANT** | **IRRELEVANT** | **DO NOT KNOW/DO NOT WANT TO ANSWER** |
| ﻿Determinants of health | 28% | 38% | 14% | 17% | 3% | 0% | 0% | 0% |
| Health economics | 14% | 38% | 31% | 14% | 3% | 0% | 0% | 0% |
| ﻿Pharmacoeconomics and Health Technology Assessment﻿ | 10% | 28% | 41% | 17% | 3% | 0% | 0% | 0% |
| Value-based healthcare | 21% | 31% | 28% | 14% | 7% | 0% | 0% | 0% |
| Evidence-based medicine and clinical studies | 17% | 45% | 24% | 10% | 3% | 0% | 0% | 0% |
| Health geopolitics and public affairs | 21% | 10% | 41% | 14% | 14% | 0% | 0% | 0% |
| Regulation in health | 31% | 34% | 28% | 7% | 0% | 0% | 0% | 0% |
| ﻿Health systems funding, financing and policies | 31% | 31% | 31% | 7% | 0% | 0% | 0% | 0% |
| Digital health, information technology and information technology systems interoperability and integration | 41% | 28% | 24% | 7% | 0% | 0% | 0% | 0% |
| Regulation and legal frameworks in digital health | 45% | 28% | 21% | 7% | 0% | 0% | 0% | 0% |
| Hospital management | 14% | 24% | 45% | 10% | 7% | 0% | 0% | 0% |
| Patents, intellectual property and legislation | 24% | 24% | 28% | 14% | 10% | 0% | 0% | 0% |
| Market research | 17% | 10% | 34% | 24% | 10% | 3% | 0% | 0% |
| Open innovation | 21% | 38% | 17% | 17% | 0% | 3% | 0% | 3% |
| Sustainability and innovation management | 21% | 21% | 31% | 24% | 3% | 0% | 0% | 0% |
| Business funding and venture capital | 17% | 24% | 24% | 28% | 3% | 3% | 0% | 0% |
| Paths to commercialization | 24% | 21% | 21% | 14% | 21% | 0% | 0% | 0% |
| Business case analysis | 21% | 10% | 31% | 28% | 10% | 0% | 0% | 0% |
| Start-up creation and management | 10% | 17% | 31% | 24% | 14% | 3% | 0% | 0% |
| Health entrepreneurship ecosystem | 17% | 24% | 38% | 17% | 3% | 0% | 0% | 0% |
| Ethics in health entrepreneurship | 34% | 21% | 14% | 24% | 3% | 3% | 0% | 0% |
| ﻿Ethical and responsible use of artificial intelligence in health | 31% | 31% | 14% | 17% | 3% | 3% | 0% | 0% |

# 2.5. Web-Delphi Round 2 distribution and analysis of results by stakeholder group

As the 'government' stakeholder group, only has one participant, analyses of group results are not applicable in this case. Analyses of group results are only conducted for 'Health industry', 'Health care providers' and 'Academia' stakeholder groups.

**Table S3** Web-Delphi Round 2 analysis of results concerning ‘Health industry’ stakeholder group (N=13 participants).

| **‘PSYCHOLOGICAL/SELF-MANAGEMENT’ E&I COMPETENCIES**  **COURSE TOPICS** | **EXTREMELY**  **RELEVANT** | **VERY STRONGLY**  **RELEVANT** | **STRONGLY**  **RELEVANT** | **MODERATELY**  **RELEVANT** | **WEAKLY**  **RELEVANT** | **VERY WEAKLY RELEVANT** | **IRRELEVANT** | **DO NOT KNOW/DO NOT WANT TO ANSWER** | **STAKEHOLDER GROUP MAJORITY OPINION*** | **STAKEHOLDER GROUP AGREEMENT ON TOPIC RELEVANCE**** |
| --- | --- | --- | --- | --- | --- | --- | --- | --- | --- | --- |
| Behavioural and cognitive biases in decision-making | 15% | 31% | 38% | 15% | 0% | 0% | 0% | 0% | **Very strongly relevant - Strongly relevant** | **Yes (85%)** |
| Know Thyself (Psychology of Personality) | 23% | 8% | 69% | 0% | 0% | 0% | 0% | 0% | **Strongly relevant** | **Yes (100%)** |
| Emotional Intelligence | 46% | 31% | 23% | 0% | 0% | 0% | 0% | 0% | **Extremely relevant - Very strongly relevant** | **Yes (100%)** |
| Mental health and well-being | 62% | 15% | 15% | 8% | 0% | 0% | 0% | 0% | **Extremely relevant** | **Yes (92%)** |
| Professional resilience | 38% | 38% | 23% | 0% | 0% | 0% | 0% | 0% | **Extremely relevant - Very strongly relevant** | **Yes (100%)** |
| Critical Thinking | 69% | 23% | 8% | 0% | 0% | 0% | 0% | 0% | **Extremely relevant** | **Yes (100%)** |
| Risk taking and dealing with failure | 54% | 46% | 0% | 0% | 0% | 0% | 0% | 0% | **Extremely relevant** | **Yes (100%)** |
| Time and stress management | 31% | 46% | 23% | 0% | 0% | 0% | 0% | 0% | **Extremely relevant - Very strongly relevant** | **Yes (100%)** |
| **‘SOCIAL/INTERPERSONAL’ E&I COMPETENCIES**  **COURSE TOPICS** | **EXTREMELY**  **RELEVANT** | **VERY STRONGLY**  **RELEVANT** | **STRONGLY**  **RELEVANT** | **MODERATELY**  **RELEVANT** | **WEAKLY**  **RELEVANT** | **VERY WEAKLY RELEVANT** | **IRRELEVANT** | **DO NOT KNOW/DO NOT WANT TO ANSWER** | **STAKEHOLDER GROUP MAJORITY OPINION*** | **STAKEHOLDER GROUP AGREEMENT ON TOPIC RELEVANCE**** |
| Leadership and management theories | 38% | 31% | 31% | 0% | 0% | 0% | 0% | 0% | **Extremely relevant - Very strongly relevant** | **Yes (100%)** |
| Public speaking and communication | 23% | 62% | 15% | 0% | 0% | 0% | 0% | 0% | **Very strongly relevant** | **Yes (100%)** |
| Media training | 0% | 31% | 31% | 23% | 15% | 0% | 0% | 0% | **Very strongly relevant - Strongly relevant** | **No (62%)** |
| Stakeholder engagement | 69% | 23% | 8% | 0% | 0% | 0% | 0% | 0% | **Extremely relevant** | **Yes (100%)** |
| Organizational behaviour and social psychology | 8% | 62% | 23% | 8% | 0% | 0% | 0% | 0% | **Very strongly relevant** | **Yes (92%)** |
| Team management and facilitation of group processes | 62% | 8% | 31% | 0% | 0% | 0% | 0% | 0% | **Extremely relevant** | **Yes (100%)** |
| Negotiation | 38% | 38% | 23% | 0% | 0% | 0% | 0% | 0% | **Extremely relevant - Very strongly relevant** | **Yes (100%)** |
| Conflict management | 38% | 38% | 23% | 0% | 0% | 0% | 0% | 0% | **Extremely relevant - Very strongly relevant** | **Yes (100%)** |
| Cross-cultural communication | 15% | 54% | 31% | 0% | 0% | 0% | 0% | 0% | **Very strongly relevant** | **Yes (100%)** |
| **‘AT THE ORHANIZATIONAL LEVEL’ E&I COMPETENCIES**  **COURSE TOPICS** | **EXTREMELY**  **RELEVANT** | **VERY STRONGLY**  **RELEVANT** | **STRONGLY**  **RELEVANT** | **MODERATELY**  **RELEVANT** | **WEAKLY**  **RELEVANT** | **VERY WEAKLY RELEVANT** | **IRRELEVANT** | **DO NOT KNOW/DO NOT WANT TO ANSWER** | **STAKEHOLDER GROUP MAJORITY OPINION*** | **STAKEHOLDER GROUP AGREEMENT ON TOPIC RELEVANCE**** |
| Organizational strategy | 38% | 62% | 0% | 0% | 0% | 0% | 0% | 0% | **Very strongly relevant** | **Yes (100%)** |
| Human resources management | 46% | 31% | 15% | 8% | 0% | 0% | 0% | 0% | **Very strongly relevant - Strongly relevant** | **Yes (92%)** |
| Change management | 38% | 62% | 0% | 0% | 0% | 0% | 0% | 0% | **Very strongly relevant** | **Yes (100%)** |
| Ethics and social responsibility | 31% | 46% | 23% | 0% | 0% | 0% | 0% | 0% | **Very strongly relevant - Strongly relevant** | **Yes (100%)** |
| Data privacy and security | 15% | 69% | 15% | 0% | 0% | 0% | 0% | 0% | **Very strongly relevant** | **Yes (100%)** |
| **‘ANALYTICAL/TECHNICAL’ E&I COMPETENCIES**  **COURSE TOPICS** | **EXTREMELY**  **RELEVANT** | **VERY STRONGLY**  **RELEVANT** | **STRONGLY**  **RELEVANT** | **MODERATELY**  **RELEVANT** | **WEAKLY**  **RELEVANT** | **VERY WEAKLY RELEVANT** | **IRRELEVANT** | **DO NOT KNOW/DO NOT WANT TO ANSWER** | **STAKEHOLDER GROUP MAJORITY OPINION*** | **STAKEHOLDER GROUP AGREEMENT ON TOPIC RELEVANCE**** |
| Project management | 31% | 38% | 31% | 0% | 0% | 0% | 0% | 0% | **Very strongly relevant - Strongly relevant** | **Yes (100%)** |
| Financial accounting and management | 15% | 8% | 38% | 38% | 0% | 0% | 0% | 0% | **Strongly relevant - Moderately relevant** | **No (62%)** |
| Business planning | 15% | 31% | 54% | 0% | 0% | 0% | 0% | 0% | **Strongly relevant** | **Yes (100%)** |
| Marketing | 31% | 0% | 46% | 23% | 0% | 0% | 0% | 0% | **Strongly relevant - Moderately relevant** | **Yes (77%)** |
| Decision analysis | 23% | 46% | 31% | 0% | 0% | 0% | 0% | 0% | **Very strongly relevant - Strongly relevant** | **Yes (100%)** |
| Systems thinking | 31% | 15% | 46% | 0% | 0% | 0% | 0% | 8% | **Very strongly relevant - Strongly relevant** | **Yes (92%)** |
| Risk management | 23% | 46% | 31% | 0% | 0% | 0% | 0% | 0% | **Very strongly relevant - Strongly relevant** | **Yes (100%)** |
| Quality management | 0% | 62% | 15% | 23% | 0% | 0% | 0% | 0% | **Very strongly relevant** | **Yes (77%)** |
| Lean management | 8% | 46% | 31% | 8% | 0% | 0% | 0% | 8% | **Very strongly relevant - Strongly relevant** | **Yes (85%)** |
| Design thinking and idea generation | 31% | 23% | 46% | 0% | 0% | 0% | 0% | 0% | **Very strongly relevant - Strongly relevant** | **Yes (100%)** |
| Data analytics, big data and artificial intelligence | 23% | 38% | 38% | 0% | 0% | 0% | 0% | 0% | **Very strongly relevant - Strongly relevant** | **Yes (100%)** |
| **‘HEALTH SYSTEM CONTEXTUAL KNOWLEDGE’ E&I COMPETENCIES**  **COURSE TOPICS** | **EXTREMELY**  **RELEVANT** | **VERY STRONGLY**  **RELEVANT** | **STRONGLY**  **RELEVANT** | **MODERATELY**  **RELEVANT** | **WEAKLY**  **RELEVANT** | **VERY WEAKLY RELEVANT** | **IRRELEVANT** | **DO NOT KNOW/DO NOT WANT TO ANSWER** | **STAKEHOLDER GROUP MAJORITY OPINION*** | **STAKEHOLDER GROUP AGREEMENT ON TOPIC RELEVANCE**** |
| ﻿Determinants of health | 23% | 62% | 8% | 8% | 0% | 0% | 0% | 0% | Very strongly relevant | **Yes (92%)** |
| Health economics | 15% | 54% | 31% | 0% | 0% | 0% | 0% | 0% | Very strongly relevant | **Yes (100%)** |
| ﻿Pharmacoeconomics and Health Technology Assessment﻿ | 8% | 46% | 46% | 0% | 0% | 0% | 0% | 0% | Very strongly relevant - Strongly relevant | **Yes (100%)** |
| Value-based healthcare | 23% | 54% | 15% | 8% | 0% | 0% | 0% | 0% | Very strongly relevant | **Yes (92%)** |
| Evidence-based medicine and clinical studies | 15% | 62% | 15% | 8% | 0% | 0% | 0% | 0% | Very strongly relevant | **Yes (92%)** |
| Health geopolitics and public affairs | 31% | 8% | 54% | 8% | 0% | 0% | 0% | 0% | Strongly relevant | **Yes (92%)** |
| Regulation in health | 31% | 54% | 8% | 8% | 0% | 0% | 0% | 0% | Very strongly relevant | **Yes (92%)** |
| ﻿Health systems funding, financing and policies | 46% | 38% | 15% | 0% | 0% | 0% | 0% | 0% | Extremely relevant - Very strongly relevant | **Yes (100%)** |
| Digital health, information technology and information technology systems interoperability and integration | 62% | 31% | 8% | 0% | 0% | 0% | 0% | 0% | Extremely relevant | **Yes (100%)** |
| Regulation and legal frameworks in digital health | 69% | 15% | 15% | 0% | 0% | 0% | 0% | 0% | Extremely relevant | **Yes (100%)** |
| Hospital management | 15% | 31% | 38% | 15% | 0% | 0% | 0% | 0% | Very strongly relevant - Strongly relevant | **Yes (85%)** |
| Patents, intellectual property and legislation | 23% | 31% | 38% | 8% | 0% | 0% | 0% | 0% | Very strongly relevant - Strongly relevant | **Yes (92%)** |
| Market research | 31% | 15% | 38% | 15% | 0% | 0% | 0% | 0% | Very strongly relevant - Strongly relevant | **Yes (85%)** |
| Open innovation | 15% | 54% | 15% | 0% | 0% | 0% | 0% | 8% | Very strongly relevant | **Yes (85%)** |
| Sustainability and innovation management | 31% | 38% | 15% | 15% | 0% | 0% | 0% | 0% | Extremely relevant - Very strongly relevant | **Yes (85%)** |
| Business funding and venture capital | 23% | 31% | 15% | 23% | 8% | 0% | 0% | 0% | Extremely relevant - Very strongly relevant | **No (69%)** |
| Paths to commercialization | 46% | 15% | 23% | 0% | 15% | 0% | 0% | 0% | Extremely relevant - Very strongly relevant | **Yes (85%)** |
| Business case analysis | 38% | 15% | 38% | 8% | 0% | 0% | 0% | 0% | Very strongly relevant - Strongly relevant | **Yes (92%)** |
| Start-up creation and management | 15% | 8% | 54% | 23% | 0% | 0% | 0% | 0% | Strongly relevant | **Yes (77%)** |
| Health entrepreneurship ecosystem | 31% | 31% | 31% | 8% | 0% | 0% | 0% | 0% | Very strongly relevant - Strongly relevant | **Yes (92%)** |
| Ethics in health entrepreneurship | 38% | 31% | 15% | 15% | 0% | 0% | 0% | 0% | Extremely relevant - Very strongly relevant | **Yes (85%)** |
| ﻿Ethical and responsible use of artificial intelligence in health | 31% | 38% | 15% | 15% | 0% | 0% | 0% | 0% | Extremely relevant - Very strongly relevant | **Yes (85%)** |

*‘Stakeholder group majority opinion’ is determined using the following criteria: When a single relevance category achieves more than 50% of responses, it is designated as the majority opinion and highlighted in dark grey. When no individual category exceeds 50%, consecutive relevance categories are sequentially combined until the cumulative percentage surpasses 50% (shown in light grey). In instances where three consecutive categories are required to achieve majority, the two categories representing lower relevance levels are selected. When four consecutive categories tie, the two central categories are considered.

**‘Stakeholder group agreement on topic relevance’ is achieved when the combined percentage of responses across the three highest relevance categories (Extremely Relevant, Very Strongly Relevant, and Strongly Relevant) equals or exceeds the predetermined 70% threshold. Topics meeting this criterion are designated "Yes," while those below the threshold are designated "No”, with the corresponding percentages shown in brackets.

**Table S4** Web-Delphi Round 2 analysis of results concerning ‘Health care providers’ stakeholder group (N=8 participants).

| **‘PSYCHOLOGICAL/SELF-MANAGEMENT’ E&I COMPETENCIES**  **COURSE TOPICS** | **EXTREMELY**  **RELEVANT** | **VERY STRONGLY**  **RELEVANT** | **STRONGLY**  **RELEVANT** | **MODERATELY**  **RELEVANT** | **WEAKLY**  **RELEVANT** | **VERY WEAKLY RELEVANT** | **IRRELEVANT** | **DO NOT KNOW/DO NOT WANT TO ANSWER** | **STAKEHOLDER GROUP MAJORITY OPINION*** | **STAKEHOLDER GROUP AGREEMENT ON TOPIC RELEVANCE**** |
| --- | --- | --- | --- | --- | --- | --- | --- | --- | --- | --- |
| Behavioural and cognitive biases in decision-making | 38% | 63% | 0% | 0% | 0% | 0% | 0% | 0% | **Very strongly relevant** | **Yes (100%)** |
| Know Thyself (Psychology of Personality) | 13% | 25% | 50% | 13% | 0% | 0% | 0% | 0% | **Strongly relevant** | **Yes (88%)** |
| Emotional Intelligence | 25% | 38% | 38% | 0% | 0% | 0% | 0% | 0% | **Very strongly relevant - Strongly relevant** | **Yes (100%)** |
| Mental health and well-being | 50% | 25% | 13% | 13% | 0% | 0% | 0% | 0% | **Extremely relevant** | **Yes (88%)** |
| Professional resilience | 25% | 38% | 25% | 13% | 0% | 0% | 0% | 0% | **Very strongly relevant - Strongly relevant** | **Yes (88%)** |
| Critical Thinking | 75% | 13% | 13% | 0% | 0% | 0% | 0% | 0% | **Extremely relevant** | **Yes (100%)** |
| Risk taking and dealing with failure | 38% | 38% | 25% | 0% | 0% | 0% | 0% | 0% | **Extremely relevant - Very strongly relevant** | **Yes (100%)** |
| Time and stress management | 38% | 13% | 38% | 13% | 0% | 0% | 0% | 0% | **Very strongly relevant - Strongly relevant** | **Yes (88%)** |
| **‘SOCIAL/INTERPERSONAL’ E&I COMPETENCIES**  **COURSE TOPICS** | **EXTREMELY**  **RELEVANT** | **VERY STRONGLY**  **RELEVANT** | **STRONGLY**  **RELEVANT** | **MODERATELY**  **RELEVANT** | **WEAKLY**  **RELEVANT** | **VERY WEAKLY RELEVANT** | **IRRELEVANT** | **DO NOT KNOW/DO NOT WANT TO ANSWER** | **STAKEHOLDER GROUP MAJORITY OPINION*** | **STAKEHOLDER GROUP AGREEMENT ON TOPIC RELEVANCE**** |
| Leadership and management theories | 38% | 13% | 38% | 13% | 0% | 0% | 0% | 0% | **Very strongly relevant - Strongly relevant** | **Yes (88%)** |
| Public speaking and communication | 13% | 25% | 50% | 13% | 0% | 0% | 0% | 0% | **Very strongly relevant - Strongly relevant** | **Yes (88%)** |
| Media training | 0% | 25% | 13% | 63% | 0% | 0% | 0% | 0% | **Strongly relevant - Moderately relevant** | **No (38%)** |
| Stakeholder engagement | 38% | 38% | 25% | 0% | 0% | 0% | 0% | 0% | **Extremely relevant - Very strongly relevant** | **Yes (100%)** |
| Organizational behaviour and social psychology | 13% | 38% | 38% | 13% | 0% | 0% | 0% | 0% | **Very strongly relevant - Strongly relevant** | **Yes (88%)** |
| Team management and facilitation of group processes | 63% | 25% | 13% | 0% | 0% | 0% | 0% | 0% | **Extremely relevant** | **Yes (100%)** |
| Negotiation | 25% | 38% | 38% | 0% | 0% | 0% | 0% | 0% | **Very strongly relevant - Strongly relevant** | **Yes (100%)** |
| Conflict management | 38% | 38% | 25% | 0% | 0% | 0% | 0% | 0% | **Extremely relevant - Very strongly relevant** | **Yes (100%)** |
| Cross-cultural communication | 13% | 13% | 50% | 25% | 0% | 0% | 0% | 0% | **Strongly relevant - Moderately relevant** | **Yes (75%)** |
| **‘AT THE ORHANIZATIONAL LEVEL’ E&I COMPETENCIES**  **COURSE TOPICS** | **EXTREMELY**  **RELEVANT** | **VERY STRONGLY**  **RELEVANT** | **STRONGLY**  **RELEVANT** | **MODERATELY**  **RELEVANT** | **WEAKLY**  **RELEVANT** | **VERY WEAKLY RELEVANT** | **IRRELEVANT** | **DO NOT KNOW/DO NOT WANT TO ANSWER** | **STAKEHOLDER GROUP MAJORITY OPINION*** | **STAKEHOLDER GROUP AGREEMENT ON TOPIC RELEVANCE**** |
| Organizational strategy | 50% | 25% | 25% | 0% | 0% | 0% | 0% | 0% | **Extremely relevant - Very strongly relevant** | **Yes (100%)** |
| Human resources management | 0% | 75% | 13% | 13% | 0% | 0% | 0% | 0% | **Very strongly relevant** | **Yes (88%)** |
| Change management | 38% | 25% | 25% | 0% | 0% | 0% | 0% | 13% | **Extremely relevant - Very strongly relevant** | **Yes (88%)** |
| Ethics and social responsibility | 50% | 25% | 25% | 0% | 0% | 0% | 0% | 0% | **Extremely relevant - Very strongly relevant** | **Yes (100%)** |
| Data privacy and security | 13% | 50% | 25% | 0% | 13% | 0% | 0% | 0% | **Very strongly relevant - Strongly relevant** | **Yes (88%)** |
| **‘ANALYTICAL/TECHNICAL’ E&I COMPETENCIES**  **COURSE TOPICS** | **EXTREMELY**  **RELEVANT** | **VERY STRONGLY**  **RELEVANT** | **STRONGLY**  **RELEVANT** | **MODERATELY**  **RELEVANT** | **WEAKLY**  **RELEVANT** | **VERY WEAKLY RELEVANT** | **IRRELEVANT** | **DO NOT KNOW/DO NOT WANT TO ANSWER** | **STAKEHOLDER GROUP MAJORITY OPINION*** | **STAKEHOLDER GROUP AGREEMENT ON TOPIC RELEVANCE**** |
| Project management | 50% | 25% | 25% | 0% | 0% | 0% | 0% | 0% | **Extremely relevant - Very strongly relevant** | **Yes (100%)** |
| Financial accounting and management | 0% | 0% | 63% | 38% | 0% | 0% | 0% | 0% | **Strongly relevant** | **No (63%)** |
| Business planning | 0% | 13% | 88% | 0% | 0% | 0% | 0% | 0% | **Strongly relevant** | **Yes (100%)** |
| Marketing | 0% | 0% | 63% | 25% | 13% | 0% | 0% | 0% | **Strongly relevant** | **No (63%)** |
| Decision analysis | 25% | 13% | 50% | 13% | 0% | 0% | 0% | 0% | **Very strongly relevant - Strongly relevant** | **Yes (88%)** |
| Systems thinking | 25% | 38% | 25% | 13% | 0% | 0% | 0% | 0% | **Very strongly relevant - Strongly relevant** | **Yes (88%)** |
| Risk management | 25% | 13% | 63% | 0% | 0% | 0% | 0% | 0% | **Strongly relevant** | **Yes (100%)** |
| Quality management | 0% | 38% | 13% | 50% | 0% | 0% | 0% | 0% | **Strongly relevant - Moderately relevant** | **No (50%)** |
| Lean management | 0% | 38% | 38% | 25% | 0% | 0% | 0% | 0% | **Very strongly relevant - Strongly relevant** | **Yes (75%)** |
| Design thinking and idea generation | 38% | 38% | 13% | 13% | 0% | 0% | 0% | 0% | **Extremely relevant - Very strongly relevant** | **Yes (88%)** |
| Data analytics, big data and artificial intelligence | 0% | 25% | 75% | 0% | 0% | 0% | 0% | 0% | **Strongly relevant** | **Yes (100%)** |
| **‘HEALTH SYSTEM CONTEXTUAL KNOWLEDGE’ E&I COMPETENCIES**  **COURSE TOPICS** | **EXTREMELY**  **RELEVANT** | **VERY STRONGLY**  **RELEVANT** | **STRONGLY**  **RELEVANT** | **MODERATELY**  **RELEVANT** | **WEAKLY**  **RELEVANT** | **VERY WEAKLY RELEVANT** | **IRRELEVANT** | **DO NOT KNOW/DO NOT WANT TO ANSWER** | **STAKEHOLDER GROUP MAJORITY OPINION*** | **STAKEHOLDER GROUP AGREEMENT ON TOPIC RELEVANCE**** |
| ﻿Determinants of health | 25% | 50% | 13% | 13% | 0% | 0% | 0% | 0% | **Extremely relevant - Very strongly relevant** | **Yes (88%)** |
| Health economics | 0% | 38% | 50% | 13% | 0% | 0% | 0% | 0% | **Very strongly relevant - Strongly relevant** | **Yes (88%)** |
| ﻿Pharmacoeconomics and Health Technology Assessment﻿ | 0% | 0% | 88% | 13% | 0% | 0% | 0% | 0% | **Strongly relevant** | **Yes (88%)** |
| Value-based healthcare | 13% | 13% | 75% | 0% | 0% | 0% | 0% | 0% | **Strongly relevant** | **Yes (100%)** |
| Evidence-based medicine and clinical studies | 0% | 63% | 38% | 0% | 0% | 0% | 0% | 0% | **Very strongly relevant** | **Yes (100%)** |
| Health geopolitics and public affairs | 0% | 0% | 75% | 0% | 25% | 0% | 0% | 0% | **Strongly relevant** | **Yes (75%)** |
| Regulation in health | 13% | 25% | 63% | 0% | 0% | 0% | 0% | 0% | **Strongly relevant** | **Yes (100%)** |
| ﻿Health systems funding, financing and policies | 0% | 25% | 75% | 0% | 0% | 0% | 0% | 0% | **Strongly relevant** | **Yes (100%)** |
| Digital health, information technology and information technology systems interoperability and integration | 38% | 13% | 50% | 0% | 0% | 0% | 0% | 0% | **Very strongly relevant - Strongly relevant** | **Yes (100%)** |
| Regulation and legal frameworks in digital health | 38% | 25% | 25% | 13% | 0% | 0% | 0% | 0% | **Extremely relevant - Very strongly relevant** | **Yes (88%)** |
| Hospital management | 13% | 38% | 38% | 13% | 0% | 0% | 0% | 0% | **Very strongly relevant - Strongly relevant** | **Yes (88%)** |
| Patents, intellectual property and legislation | 0% | 38% | 13% | 50% | 0% | 0% | 0% | 0% | **Strongly relevant - Moderately relevant** | **No (50%)** |
| Market research | 0% | 0% | 38% | 50% | 13% | 0% | 0% | 0% | **Strongly relevant - Moderately relevant** | **No (38%)** |
| Open innovation | 25% | 38% | 25% | 13% | 0% | 0% | 0% | 0% | **Very strongly relevant - Strongly relevant** | **Yes (88%)** |
| Sustainability and innovation management | 13% | 13% | 38% | 38% | 0% | 0% | 0% | 0% | **Strongly relevant - Moderately relevant** | **No (63%)** |
| Business funding and venture capital | 0% | 25% | 38% | 38% | 0% | 0% | 0% | 0% | **Strongly relevant - Moderately relevant** | **No (63%)** |
| Paths to commercialization | 0% | 13% | 38% | 13% | 38% | 0% | 0% | 0% | **Very strongly relevant - Strongly relevant - Moderately relevant** | **No (50%)** |
| Business case analysis | 0% | 0% | 38% | 50% | 13% | 0% | 0% | 0% | **Strongly relevant - Moderately relevant** | **No (38%)** |
| Start-up creation and management | 0% | 0% | 50% | 38% | 13% | 0% | 0% | 0% | **Strongly relevant - Moderately relevant** | **No (50%)** |
| Health entrepreneurship ecosystem | 0% | 13% | 63% | 25% | 0% | 0% | 0% | 0% | **Strongly relevant** | **Yes (75%)** |
| Ethics in health entrepreneurship | 25% | 25% | 13% | 38% | 0% | 0% | 0% | 0% | **Strongly relevant - Moderately relevant** | **No (63%)** |
| ﻿Ethical and responsible use of artificial intelligence in health | 38% | 25% | 13% | 25% | 0% | 0% | 0% | 0% | **Extremely relevant - Very strongly relevant** | **Yes (75%)** |

*‘Stakeholder group majority opinion’ is determined using the following criteria: When a single relevance category achieves more than 50% of responses, it is designated as the majority opinion and highlighted in dark grey. When no individual category exceeds 50%, consecutive relevance categories are sequentially combined until the cumulative percentage surpasses 50% (shown in light grey). In instances where three consecutive categories are required to achieve majority, the two categories representing lower relevance levels are selected. When four consecutive categories tie, the two central categories are considered.

**‘Stakeholder group agreement on topic relevance’ is achieved when the combined percentage of responses across the three highest relevance categories (Extremely Relevant, Very Strongly Relevant, and Strongly Relevant) equals or exceeds the predetermined 70% threshold. Topics meeting this criterion are designated "Yes," while those below the threshold are designated "No”, with the corresponding percentages shown in brackets.

**Table S5** Web-Delphi Round 2 analysis of results concerning ‘Academia’ stakeholder group (N=7 participants).

| **‘PSYCHOLOGICAL/SELF-MANAGEMENT’ E&I COMPETENCIES**  **COURSE TOPICS** | **EXTREMELY**  **RELEVANT** | **VERY STRONGLY**  **RELEVANT** | **STRONGLY**  **RELEVANT** | **MODERATELY**  **RELEVANT** | **WEAKLY**  **RELEVANT** | **VERY WEAKLY RELEVANT** | **IRRELEVANT** | **DO NOT KNOW/DO NOT WANT TO ANSWER** | **STAKEHOLDER GROUP MAJORITY OPINION*** | **STAKEHOLDER GROUP AGREEMENT ON TOPIC RELEVANCE**** |
| --- | --- | --- | --- | --- | --- | --- | --- | --- | --- | --- |
| Behavioural and cognitive biases in decision-making | 29% | 29% | 29% | 0% | 14% | 0% | 0% | 0% | **Very strongly relevant - Strongly relevant** | **Yes (86%)** |
| Know Thyself (Psychology of Personality) | 14% | 0% | 57% | 14% | 0% | 14% | 0% | 0% | **Strongly relevant** | **Yes (71%)** |
| Emotional Intelligence | 29% | 57% | 14% | 0% | 0% | 0% | 0% | 0% | **Very strongly relevant** | **Yes (100%)** |
| Mental health and well-being | 71% | 14% | 14% | 0% | 0% | 0% | 0% | 0% | **Extremely relevant** | **Yes (100%)** |
| Professional resilience | 29% | 43% | 14% | 0% | 0% | 14% | 0% | 0% | **Extremely relevant - Very strongly relevant** | **Yes (86%)** |
| Critical Thinking | 57% | 43% | 0% | 0% | 0% | 0% | 0% | 0% | **Extremely relevant** | **Yes (100%)** |
| Risk taking and dealing with failure | 29% | 43% | 29% | 0% | 0% | 0% | 0% | 0% | **Very strongly relevant - Strongly relevant** | **Yes (100%)** |
| Time and stress management | 43% | 0% | 57% | 0% | 0% | 0% | 0% | 0% | **Strongly relevant** | **Yes (100%)** |
| **‘SOCIAL/INTERPERSONAL’ E&I COMPETENCIES**  **COURSE TOPICS** | **EXTREMELY**  **RELEVANT** | **VERY STRONGLY**  **RELEVANT** | **STRONGLY**  **RELEVANT** | **MODERATELY**  **RELEVANT** | **WEAKLY**  **RELEVANT** | **VERY WEAKLY RELEVANT** | **IRRELEVANT** | **DO NOT KNOW/DO NOT WANT TO ANSWER** | **STAKEHOLDER GROUP MAJORITY OPINION*** | **STAKEHOLDER GROUP AGREEMENT ON TOPIC RELEVANCE**** |
| Leadership and management theories | 29% | 0% | 43% | 14% | 14% | 0% | 0% | 0% | **Strongly relevant - Moderately relevant** | **Yes (71%)** |
| Public speaking and communication | 14% | 57% | 14% | 14% | 0% | 0% | 0% | 0% | **Very strongly relevant** | **Yes (86%)** |
| Media training | 0% | 0% | 71% | 0% | 29% | 0% | 0% | 0% | **Strongly relevant** | **Yes (71%)** |
| Stakeholder engagement | 57% | 0% | 14% | 29% | 0% | 0% | 0% | 0% | **Extremely relevant** | **Yes (71%)** |
| Organizational behaviour and social psychology | 0% | 43% | 29% | 0% | 29% | 0% | 0% | 0% | **Very strongly relevant - Strongly relevant** | **Yes (71%)** |
| Team management and facilitation of group processes | 57% | 14% | 29% | 0% | 0% | 0% | 0% | 0% | **Extremely relevant** | **Yes (100%)** |
| Negotiation | 14% | 14% | 71% | 0% | 0% | 0% | 0% | 0% | **Strongly relevant** | **Yes (100%)** |
| Conflict management | 57% | 43% | 0% | 0% | 0% | 0% | 0% | 0% | **Extremely relevant** | **Yes (100%)** |
| Cross-cultural communication | 0% | 29% | 57% | 14% | 0% | 0% | 0% | 0% | **Strongly relevant** | **Yes (86%)** |
| **‘AT THE ORHANIZATIONAL LEVEL’ E&I COMPETENCIES**  **COURSE TOPICS** | **EXTREMELY**  **RELEVANT** | **VERY STRONGLY**  **RELEVANT** | **STRONGLY**  **RELEVANT** | **MODERATELY**  **RELEVANT** | **WEAKLY**  **RELEVANT** | **VERY WEAKLY RELEVANT** | **IRRELEVANT** | **DO NOT KNOW/DO NOT WANT TO ANSWER** | **STAKEHOLDER GROUP MAJORITY OPINION*** | **STAKEHOLDER GROUP AGREEMENT ON TOPIC RELEVANCE**** |
| Organizational strategy | 14% | 57% | 0% | 29% | 0% | 0% | 0% | 0% | **Very strongly relevant** | **Yes (71%)** |
| Human resources management | 0% | 100% | 0% | 0% | 0% | 0% | 0% | 0% | **Very strongly relevant** | **Yes (100%)** |
| Change management | 14% | 71% | 14% | 0% | 0% | 0% | 0% | 0% | **Very strongly relevant** | **Yes (100%)** |
| Ethics and social responsibility | 43% | 29% | 0% | 14% | 14% | 0% | 0% | 0% | **Extremely relevant - Very strongly relevant** | **Yes (71%)** |
| Data privacy and security | 14% | 57% | 14% | 0% | 14% | 0% | 0% | 0% | **Very strongly relevant** | **Yes (86%)** |
| **‘ANALYTICAL/TECHNICAL’ E&I COMPETENCIES**  **COURSE TOPICS** | **EXTREMELY**  **RELEVANT** | **VERY STRONGLY**  **RELEVANT** | **STRONGLY**  **RELEVANT** | **MODERATELY**  **RELEVANT** | **WEAKLY**  **RELEVANT** | **VERY WEAKLY RELEVANT** | **IRRELEVANT** | **DO NOT KNOW/DO NOT WANT TO ANSWER** | **STAKEHOLDER GROUP MAJORITY OPINION*** | **STAKEHOLDER GROUP AGREEMENT ON TOPIC RELEVANCE**** |
| Project management | 14% | 57% | 29% | 0% | 0% | 0% | 0% | 0% | **Very strongly relevant** | **Yes (100%)** |
| Financial accounting and management | 0% | 14% | 71% | 14% | 0% | 0% | 0% | 0% | **Strongly relevant** | **Yes (86%)** |
| Business planning | 0% | 29% | 71% | 0% | 0% | 0% | 0% | 0% | **Strongly relevant** | **Yes (100%)** |
| Marketing | 14% | 0% | 57% | 14% | 14% | 0% | 0% | 0% | **Strongly relevant** | **Yes (71%)** |
| Decision analysis | 0% | 43% | 29% | 29% | 0% | 0% | 0% | 0% | **Very strongly relevant - Strongly relevant** | **Yes (71%)** |
| Systems thinking | 14% | 14% | 43% | 14% | 14% | 0% | 0% | 0% | **Strongly relevant - Moderately relevant** | **Yes (71%)** |
| Risk management | 0% | 57% | 29% | 14% | 0% | 0% | 0% | 0% | **Very strongly relevant** | **Yes (86%)** |
| Quality management | 0% | 43% | 29% | 29% | 0% | 0% | 0% | 0% | **Very strongly relevant - Strongly relevant** | **Yes (71%)** |
| Lean management | 0% | 29% | 29% | 14% | 29% | 0% | 0% | 0% | **Very strongly relevant - Strongly relevant** | **Yes (57%)** |
| Design thinking and idea generation | 0% | 14% | 71% | 0% | 14% | 0% | 0% | 0% | **Strongly relevant** | **Yes (86%)** |
| Data analytics, big data and artificial intelligence | 29% | 0% | 71% | 0% | 0% | 0% | 0% | 0% | **Strongly relevant** | **Yes (100%)** |
| **‘HEALTH SYSTEM CONTEXTUAL KNOWLEDGE’ E&I COMPETENCIES**  **COURSE TOPICS** | **EXTREMELY**  **RELEVANT** | **VERY STRONGLY**  **RELEVANT** | **STRONGLY**  **RELEVANT** | **MODERATELY**  **RELEVANT** | **WEAKLY**  **RELEVANT** | **VERY WEAKLY RELEVANT** | **IRRELEVANT** | **DO NOT KNOW/DO NOT WANT TO ANSWER** | **STAKEHOLDER GROUP MAJORITY OPINION*** | **STAKEHOLDER GROUP AGREEMENT ON TOPIC RELEVANCE**** |
| ﻿Determinants of health | 14% | 29% | 29% | 14% | 14% | 0% | 0% | 0% | **Very strongly relevant - Strongly relevant** | **Yes (71%)** |
| Health economics | 14% | 43% | 14% | 14% | 14% | 0% | 0% | 0% | **Very strongly relevant - Strongly relevant** | **Yes (71%)** |
| ﻿Pharmacoeconomics and Health Technology Assessment﻿ | 14% | 29% | 29% | 29% | 0% | 0% | 0% | 0% | **Very strongly relevant - Strongly relevant** | **Yes (71%)** |
| Value-based healthcare | 14% | 29% | 29% | 14% | 14% | 0% | 0% | 0% | **Very strongly relevant - Strongly relevant** | **Yes (71%)** |
| Evidence-based medicine and clinical studies | 29% | 29% | 29% | 14% | 0% | 0% | 0% | 0% | **Very strongly relevant - Strongly relevant** | **Yes (86%)** |
| Health geopolitics and public affairs | 14% | 0% | 57% | 29% | 0% | 0% | 0% | 0% | **Strongly relevant** | **Yes (71%)** |
| Regulation in health | 43% | 29% | 29% | 0% | 0% | 0% | 0% | 0% | **Extremely relevant - Very strongly relevant** | **Yes (100%)** |
| ﻿Health systems funding, financing and policies | 43% | 29% | 14% | 14% | 0% | 0% | 0% | 0% | **Extremely relevant - Very strongly relevant** | **Yes (86%)** |
| Digital health, information technology and information technology systems interoperability and integration | 43% | 14% | 43% | 0% | 0% | 0% | 0% | 0% | **Very strongly relevant - Strongly relevant** | **Yes (100%)** |
| Regulation and legal frameworks in digital health | 43% | 29% | 14% | 14% | 0% | 0% | 0% | 0% | **Extremely relevant - Very strongly relevant** | **Yes (86%)** |
| Hospital management | 14% | 0% | 71% | 14% | 0% | 0% | 0% | 0% | **Strongly relevant** | **Yes (86%)** |
| Patents, intellectual property and legislation | 14% | 29% | 29% | 0% | 29% | 0% | 0% | 0% | **Very strongly relevant - Strongly relevant** | **Yes (71%)** |
| Market research | 0% | 29% | 29% | 14% | 14% | 14% | 0% | 0% | **Very strongly relevant - Strongly relevant** | **No (57%)** |
| Open innovation | 14% | 43% | 0% | 29% | 0% | 14% | 0% | 0% | **Extremely relevant - Very strongly relevant** | **No (57%)** |
| Sustainability and innovation management | 0% | 14% | 57% | 14% | 14% | 0% | 0% | 0% | **Strongly relevant** | **No (71%)** |
| Business funding and venture capital | 14% | 29% | 14% | 29% | 0% | 14% | 0% | 0% | **Strongly relevant - Moderately relevant** | **No (57%)** |
| Paths to commercialization | 14% | 29% | 14% | 29% | 14% | 0% | 0% | 0% | **Strongly relevant - Moderately relevant** | **No (57%)** |
| Business case analysis | 0% | 0% | 43% | 43% | 14% | 0% | 0% | 0% | **Strongly relevant - Moderately relevant** | **No (43%)** |
| Start-up creation and management | 0% | 14% | 43% | 14% | 29% | 0% | 0% | 0% | **Strongly relevant - Moderately relevant** | **No (57%)** |
| Health entrepreneurship ecosystem | 0% | 43% | 29% | 14% | 14% | 0% | 0% | 0% | **Very strongly relevant - Strongly relevant** | **Yes (71%)** |
| Ethics in health entrepreneurship | 43% | 14% | 14% | 29% | 0% | 0% | 0% | 0% | **Extremely relevant - Very strongly relevant** | **Yes (71%)** |
| ﻿Ethical and responsible use of artificial intelligence in health | 29% | 43% | 0% | 29% | 0% | 0% | 0% | 0% | **Extremely relevant - Very strongly relevant** | **Yes (71%)** |

*‘Stakeholder group majority opinion’ is determined using the following criteria: When a single relevance category achieves more than 50% of responses, it is designated as the majority opinion and highlighted in dark grey. When no individual category exceeds 50%, consecutive relevance categories are sequentially combined until the cumulative percentage surpasses 50% (shown in light grey). In instances where three consecutive categories are required to achieve majority, the two categories representing lower relevance levels are selected. When four consecutive categories tie, the two central categories are considered.

**‘Stakeholder group agreement on topic relevance’ is achieved when the combined percentage of responses across the three highest relevance categories (Extremely Relevant, Very Strongly Relevant, and Strongly Relevant) equals or exceeds the predetermined 70% threshold. Topics meeting this criterion are designated "Yes," while those below the threshold are designated "No”, with the corresponding percentages shown in brackets.

**Table S5** Web-Delphi Round 2 distribution of results concerning ‘Government’ stakeholder group (N=1 participant).

| **‘PSYCHOLOGICAL/SELF-MANAGEMENT’ E&I COMPETENCIES**  **COURSE TOPICS** | **EXTREMELY**  **RELEVANT** | **VERY STRONGLY**  **RELEVANT** | **STRONGLY**  **RELEVANT** | **MODERATELY**  **RELEVANT** | **WEAKLY**  **RELEVANT** | **VERY WEAKLY RELEVANT** | **IRRELEVANT** | **DO NOT KNOW/DO NOT WANT TO ANSWER** |
| --- | --- | --- | --- | --- | --- | --- | --- | --- |
| Behavioural and cognitive biases in decision-making | 0% | 100% | 0% | 0% | 0% | 0% | 0% | 0% |
| Know Thyself (Psychology of Personality) | 0% | 0% | 100% | 0% | 0% | 0% | 0% | 0% |
| Emotional Intelligence | 0% | 100% | 0% | 0% | 0% | 0% | 0% | 0% |
| Mental health and well-being | 100% | 0% | 0% | 0% | 0% | 0% | 0% | 0% |
| Professional resilience | 100% | 0% | 0% | 0% | 0% | 0% | 0% | 0% |
| Critical Thinking | 100% | 0% | 0% | 0% | 0% | 0% | 0% | 0% |
| Risk taking and dealing with failure | 0% | 100% | 0% | 0% | 0% | 0% | 0% | 0% |
| Time and stress management | 100% | 0% | 0% | 0% | 0% | 0% | 0% | 0% |
| **‘SOCIAL/INTERPERSONAL’ E&I COMPETENCIES**  **COURSE TOPICS** | **EXTREMELY**  **RELEVANT** | **VERY STRONGLY**  **RELEVANT** | **STRONGLY**  **RELEVANT** | **MODERATELY**  **RELEVANT** | **WEAKLY**  **RELEVANT** | **VERY WEAKLY RELEVANT** | **IRRELEVANT** | **DO NOT KNOW/DO NOT WANT TO ANSWER** |
| Leadership and management theories | 0% | 100% | 0% | 0% | 0% | 0% | 0% | 0% |
| Public speaking and communication | 0% | 100% | 0% | 0% | 0% | 0% | 0% | 0% |
| Media training | 0% | 0% | 100% | 0% | 0% | 0% | 0% | 0% |
| Stakeholder engagement | 100% | 0% | 0% | 0% | 0% | 0% | 0% | 0% |
| Organizational behaviour and social psychology | 0% | 0% | 100% | 0% | 0% | 0% | 0% | 0% |
| Team management and facilitation of group processes | 0% | 100% | 0% | 0% | 0% | 0% | 0% | 0% |
| Negotiation | 0% | 0% | 100% | 0% | 0% | 0% | 0% | 0% |
| Conflict management | 0% | 100% | 0% | 0% | 0% | 0% | 0% | 0% |
| Cross-cultural communication | 0% | 0% | 100% | 0% | 0% | 0% | 0% | 0% |
| **‘AT THE ORHANIZATIONAL LEVEL’ E&I COMPETENCIES**  **COURSE TOPICS** | **EXTREMELY**  **RELEVANT** | **VERY STRONGLY**  **RELEVANT** | **STRONGLY**  **RELEVANT** | **MODERATELY**  **RELEVANT** | **WEAKLY**  **RELEVANT** | **VERY WEAKLY RELEVANT** | **IRRELEVANT** | **DO NOT KNOW/DO NOT WANT TO ANSWER** |
| Organizational strategy | 0% | 100% | 0% | 0% | 0% | 0% | 0% | 0% |
| Human resources management | 0% | 100% | 0% | 0% | 0% | 0% | 0% | 0% |
| Change management | 0% | 100% | 0% | 0% | 0% | 0% | 0% | 0% |
| Ethics and social responsibility | 0% | 100% | 0% | 0% | 0% | 0% | 0% | 0% |
| Data privacy and security | 100% | 0% | 0% | 0% | 0% | 0% | 0% | 0% |
| **‘ANALYTICAL/TECHNICAL’ E&I COMPETENCIES**  **COURSE TOPICS** | **EXTREMELY**  **RELEVANT** | **VERY STRONGLY**  **RELEVANT** | **STRONGLY**  **RELEVANT** | **MODERATELY**  **RELEVANT** | **WEAKLY**  **RELEVANT** | **VERY WEAKLY RELEVANT** | **IRRELEVANT** | **DO NOT KNOW/DO NOT WANT TO ANSWER** |
| Project management | 0% | 100% | 0% | 0% | 0% | 0% | 0% | 0% |
| Financial accounting and management | 0% | 0% | 100% | 0% | 0% | 0% | 0% | 0% |
| Business planning | 0% | 0% | 100% | 0% | 0% | 0% | 0% | 0% |
| Marketing | 0% | 0% | 0% | 100% | 0% | 0% | 0% | 0% |
| Decision analysis | 0% | 0% | 100% | 0% | 0% | 0% | 0% | 0% |
| Systems thinking | 0% | 100% | 0% | 0% | 0% | 0% | 0% | 0% |
| Risk management | 0% | 100% | 0% | 0% | 0% | 0% | 0% | 0% |
| Quality management | 0% | 100% | 0% | 0% | 0% | 0% | 0% | 0% |
| Lean management | 0% | 100% | 0% | 0% | 0% | 0% | 0% | 0% |
| Design thinking and idea generation | 0% | 0% | 100% | 0% | 0% | 0% | 0% | 0% |
| Data analytics, big data and artificial intelligence | 0% | 0% | 100% | 0% | 0% | 0% | 0% | 0% |
| **‘HEALTH SYSTEM CONTEXTUAL KNOWLEDGE’ E&I COMPETENCIES**  **COURSE TOPICS** | **EXTREMELY**  **RELEVANT** | **VERY STRONGLY**  **RELEVANT** | **STRONGLY**  **RELEVANT** | **MODERATELY**  **RELEVANT** | **WEAKLY**  **RELEVANT** | **VERY WEAKLY RELEVANT** | **IRRELEVANT** | **DO NOT KNOW/DO NOT WANT TO ANSWER** |
| ﻿Determinants of health | 0% | 100% | 0% | 0% | 0% | 0% | 0% | 0% |
| Health economics | 0% | 0% | 100% | 0% | 0% | 0% | 0% | 0% |
| ﻿Pharmacoeconomics and Health Technology Assessment﻿ | 0% | 0% | 100% | 0% | 0% | 0% | 0% | 0% |
| Value-based healthcare | 0% | 0% | 100% | 0% | 0% | 0% | 0% | 0% |
| Evidence-based medicine and clinical studies | 0% | 100% | 0% | 0% | 0% | 0% | 0% | 0% |
| Health geopolitics and public affairs | 0% | 0% | 0% | 100% | 0% | 0% | 0% | 0% |
| Regulation in health | 100% | 0% | 0% | 0% | 0% | 0% | 0% | 0% |
| ﻿Health systems funding, financing and policies | 0% | 0% | 100% | 0% | 0% | 0% | 0% | 0% |
| Digital health, information technology and information technology systems interoperability and integration | 0% | 100% | 0% | 0% | 0% | 0% | 0% | 0% |
| Regulation and legal frameworks in digital health | 0% | 100% | 0% | 0% | 0% | 0% | 0% | 0% |
| Hospital management | 0% | 0% | 100% | 0% | 0% | 0% | 0% | 0% |
| Patents, intellectual property and legislation | 0% | 0% | 100% | 0% | 0% | 0% | 0% | 0% |
| Market research | 0% | 0% | 100% | 0% | 0% | 0% | 0% | 0% |
| Open innovation | 0% | 100% | 0% | 0% | 0% | 0% | 0% | 0% |
| Sustainability and innovation management | 0% | 0% | 100% | 0% | 0% | 0% | 0% | 0% |
| Business funding and venture capital | 0% | 0% | 100% | 0% | 0% | 0% | 0% | 0% |
| Paths to commercialization | 0% | 100% | 0% | 0% | 0% | 0% | 0% | 0% |
| Business case analysis | 0% | 0% | 100% | 0% | 0% | 0% | 0% | 0% |
| Start-up creation and management | 0% | 0% | 100% | 0% | 0% | 0% | 0% | 0% |
| Health entrepreneurship ecosystem | 0% | 0% | 100% | 0% | 0% | 0% | 0% | 0% |
| Ethics in health entrepreneurship | 0% | 100% | 0% | 0% | 0% | 0% | 0% | 0% |
| ﻿Ethical and responsible use of artificial intelligence in health | 0% | 100% | 0% | 0% | 0% | 0% | 0% | 0% |

# 2.6. Opinion changes between Delphi rounds 1 and 2

**Table S7** – Number of opinion changes by course topic and stakeholder group between Delphi rounds 1 and 2.

|  | **Health industry (N=13)** | **Health care providers (N=8)** | **Academia**  **(N=7)** | **Government (N=1)** | **Total opinion changes per topic (all panel)** |
| --- | --- | --- | --- | --- | --- |
| **‘PSYCHOLOGICAL/SELF-MANAGEMENT’ E&I**  **COURSE TOPICS** |  |  |  |  |  |
| Behavioural and cognitive biases in decision-making | 2 | 0 | 1 | 0 | **3** |
| Know Thyself (Psychology of Personality) | 0 | 2 | 0 | 1 | **3** |
| Emotional Intelligence | 0 | 2 | 1 | 0 | **3** |
| Mental health and well-being | 3 | 2 | 0 | 0 | **5** |
| Professional resilience | 1 | 1 | 1 | 0 | **3** |
| Critical Thinking | 2 | 0 | 2 | 0 | **4** |
| Risk taking and dealing with failure | 2 | 1 | 0 | 0 | **3** |
| Time and stress management | 1 | 1 | 1 | 0 | **3** |
| **Sub-total opinion changes by stakeholder group** | **11** | **9** | **6** | **1** |  |
| **‘SOCIAL/INTERPERSONAL’ E&I**  **COURSE TOPICS** |  |  |  |  |  |
| Leadership and management theories | 2 | 2 | 3 | 0 | **7** |
| Public speaking and communication | 1 | 0 | 1 | 0 | **2** |
| Media training | 1 | 1 | 1 | 0 | **3** |
| Stakeholder engagement | 3 | 2 | 1 | 0 | **6** |
| Organizational behaviour and social psychology | 4 | 0 | 3 | 0 | **7** |
| Team management and facilitation of group processes | 1 | 1 | 2 | 1 | **5** |
| Negotiation | 0 | 0 | 1 | 0 | **1** |
| Conflict management | 1 | 1 | 0 | 1 | **3** |
| Cross-cultural communication | 1 | 1 | 1 | 0 | **3** |
| **Sub-total opinion changes by stakeholder group** | **14** | **8** | **13** | **2** |  |
| **‘AT THE ORGANIZATIONAL LEVEL’ E&I**  **COURSE TOPICS** |  |  |  |  |  |
| Organizational strategy | 2 | 2 | 3 | 0 | **7** |
| Human resources management | 1 | 0 | 1 | 0 | **2** |
| Change management | 1 | 1 | 1 | 0 | **3** |
| Ethics and social responsibility | 3 | 2 | 1 | 0 | **6** |
| Data privacy and security | 4 | 0 | 3 | 0 | **4** |
| **Sub-total opinion changes by stakeholder group** | **11** | **5** | **9** | **0** |  |
| **‘ANALYTICAL/TECHNICAL’ E&I**  **COURSE TOPICS** |  |  |  |  |  |
| Project management | 2 | 0 | 1 | 0 | **3** |
| Financial accounting and management | 3 | 0 | 1 | 0 | **4** |
| Business planning | 2 | 1 | 2 | 0 | **5** |
| Marketing | 1 | 2 | 1 | 0 | **4** |
| Decision analysis | 0 | 1 | 1 | 0 | **2** |
| Systems thinking | 0 | 1 | 2 | 0 | **3** |
| Risk management | 2 | 1 | 2 | 0 | **5** |
| Quality management | 1 | 0 | 3 | 0 | **4** |
| Lean management | 1 | 2 | 3 | 0 | **6** |
| Design thinking and idea generation | 2 | 1 | 2 | 0 | **5** |
| Data analytics, big data and artificial intelligence | 1 | 1 | 1 | 0 | **3** |
| **Sub-total opinion changes by stakeholder group** | **15** | **10** | **19** | **0** |  |
| **‘HEALTH SYSTEM CONTEXTUAL KNOWLEDGE’ E&I COMPETENCIES**  **COURSE TOPICS** |  |  |  |  |  |
| ﻿Determinants of health | 2 | 1 | 1 | 1 | **5** |
| Health economics | 2 | 2 | 0 | 0 | **4** |
| ﻿Pharmacoeconomics and Health Technology Assessment﻿ | 3 | 0 | 1 | 1 | **5** |
| Value-based healthcare | 2 | 2 | 1 | 0 | **5** |
| Evidence-based medicine and clinical studies | 3 | 1 | 1 | 0 | **5** |
| Health geopolitics and public affairs | 2 | 1 | 3 | 0 | **6** |
| Regulation in health | 1 | 1 | 1 | 1 | **4** |
| ﻿Health systems funding, financing and policies | 1 | 0 | 0 | 0 | **1** |
| Digital health, information technology and information technology systems interoperability and integration | 3 | 2 | 0 | 1 | **6** |
| Regulation and legal frameworks in digital health | 1 | 1 | 1 | 0 | **3** |
| Hospital management | 3 | 1 | 1 | 0 | **5** |
| Patents, intellectual property and legislation | 1 | 1 | 2 | 0 | **4** |
| Market research | 1 | 1 | 1 | 0 | **3** |
| Open innovation | 2 | 1 | 1 | 1 | **6** |
| Sustainability and innovation management | 1 | 0 | 1 | 0 | **2** |
| Business funding and venture capital | 0 | 0 | 1 | 0 | **1** |
| Paths to commercialization | 1 | 0 | 0 | 0 | **1** |
| Business case analysis | 1 | 2 | 1 | 0 | **4** |
| Start-up creation and management | 5 | 1 | 1 | 0 | **7** |
| Health entrepreneurship ecosystem | 1 | 0 | 1 | 0 | **2** |
| Ethics in health entrepreneurship | 2 | 1 | 1 | 1 | **5** |
| ﻿Ethical and responsible use of artificial intelligence in health | 1 | 2 | 1 | 0 | **3** |
| **Sub-total opinion changes by stakeholder group** | **39** | **21** | **21** | **6** |  |
| **Total opinion changes** | **90** | **53** | **68** | **9** | **220** |
| **Number of possible opinion changes** | **770** | **440** | **385** | **NA** | **1595** |
| **Rate of opinion change (%)** | **12%** | **12%** | **18%** | **NA** | **14%** |

Total opinion changes is the sum of all opinion changes across all topics for that group. Number of possible changes in stakeholder group = Number of participants in stakeholder group * Number of topics. Rate of opinion change (%) = Total number of opinion changes in stakeholder group / Total possible changes in stakeholder group. Stakeholder group analyses were conducted only for groups with at least 7 participants to ensure meaningful group-level comparisons, resulting in the exclusion of the ‘Government’ stakeholder group (N=1). NA – Not Applied.

# 2.7. Results of the feedback survey (following completion of Round 2 of the web-Delphi process)

**Table S9** - Results of the feedback survey (following completion of Round 2 of the web-Delphi process).

| **To what extent do you agree with the following statement:** | **Strongly Agree** | **Agree** | **Neither Agree nor Disagree** | **Disagree** | **Strongly Disagree** | **Did not answer** | **N =29** | **%** |
| --- | --- | --- | --- | --- | --- | --- | --- | --- |
| “I clearly understood what I needed to do and the facilitation team provided clear information on the tasks to be performed.” | 10 | 17 | 2 | 0 | 0 | 0 | **29** | **100%** |
| “The design of the platform helped me understand the tasks at hand and helped me to share my views on the questions being addressed.” | 12 | 12 | 2 | 2 | 0 | 1 | **28** | **97%** |
| “The aggregated statistics of all participants’ answers helped me reflect more thoroughly on my previous response.” | 12 | 9 | 3 | 3 | 0 | 2 | **27** | **93%** |

The middle cells pertain to the number of participants providing his/her level of agreement with each statement (in rows) choosing one category from the 5-point agreement scale (in columns). The final columns of the table correspond to the total number of participants answering to each statement (in absolute terms and percentage).
